# Homo- Versus Hetero- [2+2+2] Rhodium-Catalyzed Cycloaddition: Effect of a Self-Assembled Capsule on the Catalytic Outcome

**DOI:** 10.3390/molecules30143052

**Published:** 2025-07-21

**Authors:** Maxime Steinmetz, David Sémeril

**Affiliations:** Synthèse Organométallique et Catalyse, UMR-CNRS 7177 Institut de Chimie de Strasbourg, Strasbourg University, 67008 Strasbourg, France; maxime.steinmetz@etu.unistra.fr

**Keywords:** rhodium, resorcin[4]arene, self-assembled capsule, host–guest chemistry, confined catalysis, [2+2+2] cycloaddition

## Abstract

The cationic chloro-*P*-{[4-(diphenylphosphanyl)phenyl]-*N*,*N*-dimethylmethanammonio(norbornadiene)rhodium(I) complex was encapsulated inside a self-assembled hexameric capsule. This capsule was obtained through a reaction involving 2,8,14,20-tetra-undecyl-resorcin[4]arene and water in chloroform. The formation of an inclusion complex was deduced from a combination of spectral measurements (UV-visible spectroscopy, ^1^H, ^31^P{^1^H} NMR and DOSY). The rhodium complex was evaluated in the [2+2+2] cycloaddition between *N*,*N*-dipropargyl-*p*-toluenesulfonamide and arylacetylene derivatives. In the presence of two equivalents of arylacetylenes in water-saturated chloroform at 60 °C for 24 h, the 4-methyl-*N*-(prop-2-yn-1-yl)-*N*-((2-tosylisoindolin-5-yl)methyl)benzenesulfonamide, the homocycloaddition product of 1,6-diyne is predominantly formed. In the presence of the supramolecular capsule, a selectivity inversion in favor of 5-aryl-2-tosylisoindoline is observed, with heterocycloaddition products formed in proportions between 53 and 69%.

## 1. Introduction

Reactivity and selectivity have always been problems in catalytic reactions. Improving the performance of catalytic systems takes time and involves the development of new catalysts. Drawing inspiration from nature, particularly enzymes, the use of confined spaces has emerged as a promising solution for enhancing the efficiency of catalytic systems, especially in cases where targeted reactions result in the formation of multiple products. Indeed, the confinement of a species can induce a modification of the reaction products in relation to free systems, resulting in some cases in a more active system, better selectivity and even the formation of new products [1,2,3,4].

In this context, the integration of supramolecular capsules into homogeneous catalysis has emerged as an innovative approach. These capsules, which are formed by self-assembly, have the capacity to encapsulate substrates or catalysts, thereby creating a well-defined microenvironment. These structures have been shown to mimic the function of enzymes, thereby enabling the local accumulation of reagents, control of reactivity and even acceleration or direction of specific chemical transformations through stabilization of the transition state [5,6,7,8,9,10,11,12].

**Figure 1:**
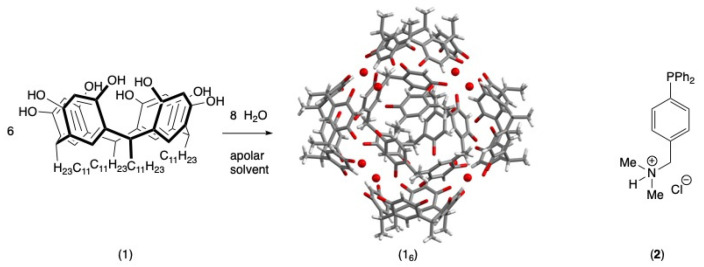
Formation of the self-assembled capsule **1**_6_ and the 4-(diphenylphosphanyl)-phenyl]-*N*,*N*-dimethylmethanamine (**2**) employed in the present study.

One of the most widely used capsules, which was discovered by Atwood, consists of six units of 2,8,14,20-tetra-undecyl-resorcin[4]arene (**1**) and eight water molecules (Figure 1) [13]. The process of host diffusion within the hexameric capsule is achieved by the temporary dissociation of a resorcinarene subunit, thereby exposing the internal cavity to substrates in the solution [14]. The encapsulation of metal complexes within **1**_6_ is predominantly predicated on the positive charge carried by the metal center or a ligand [15,16,17,18]. While this method has proven effective, its applications in catalysis are limited. The objective of this study is to utilize a readily available phosphine that can be rendered cationic. Consequently, the triphenylphosphine derivative **2** was selected for further investigation (Figure 1). The phosphorus atom facilitates coordination with a metal center, while the amine provides a cationic charge precursor site through protonation. This positive charge should facilitate π-cation interactions with the inner aromatic walls of the cavity, thereby immobilizing the complex within the capsule, as reported for numerous organometallic complexes [15,16,17,18,19,20,21,22,23,24,25,26].

**Scheme 1:**
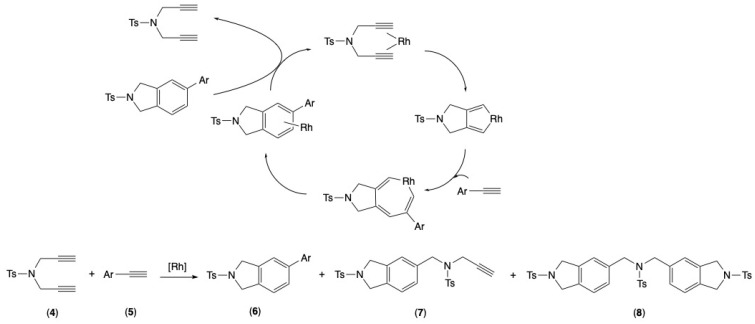
Rhodium-catalyzed [2+2+2] cycloaddition of *N*,*N*-dipropargyl-*p*-toluenesulfonamide (**4**) and arylacetylene **5**.

The rhodium(I) complex [RhCl(**2**)(nbd)] (**3**; nbd = bicyclo[2.2.1]hepta-2,5-diene) will undergo testing in the [2+2+2] cycloaddition of alkynes [27,28,29,30,31,32] in the supramolecular host. For this cycloaddition, it can be seen that the generally used ligands of choice are mono- or bis-triarylphosphines, such as triphenylphosphine and (biphenyl-2,2′-diyl)bis(diphenylphosphine) (BIPHEP) derivatives. As illustrated in Scheme 1, the reaction of *N*,*N*-dipropargyl-*p*-toluenesulfonamide (**4**) with arylacetylene **5** results in the formation of three distinct products. It was determined that these products correspond to the heterocycloaddition product **6**, as well as to mono- and bis-homocycloaddition products **7** and **8**, respectively.

In the case of phenylacetylene (**5a**), the formation of the product of interest (**6a**) has been described in the literature as catalyzed by rhodium complexes. For instance, Wu, Zhu and co-workers reported the formation of product **6a** in a 52% yield using 5 mol % of the dimeric [Rh_2_(OAc)_2_] complex in 1,2-dichloroethane at 80 °C for 24 h. However, the employment of five equivalents of phenylacetylene **5a** is imperative to facilitate the heterocycloaddition reaction [33]. On the other hand, Wu and co-workers explored the synthesis of **6a** catalyzed by the commercially available [Rh(cod)Cl]_2_ complex (1 mol %; cod = 1,5-octadiene). After one hour in a mixture of water/THF (1:1) at room temperature, the targeted product, **6a**, was isolated in a 97% yield using only two equivalents of phenylacetylene **5a** [34]. Cobalt complexes have also been shown to function as catalysts; however, the problem of the substoichiometric use of monoalkyne **5a** has been observed, with values ranging from 4 to 5 equivalents [35,36]. In the recent literature, iron- and nickel-based catalysts have been described for this reaction. The selectivity for the desired product, **6a**, is optimized with minimal monoalkyne loading (ranging from 1 to 1.5 equivalents). However, it is imperative to note that achieving a catalyst loading of 10% is necessary to ensure optimal performance [37,38,39].

It is hypothesized that the alkyne trimerization reaction, when carried out in a confined space, will modify the product distribution in favor of the sterically less bulky product of interest, **6a**. This hypothesis is based on our experience in catalysis with inclusion complexes generated from neutral complexes and supramolecular capsule **1**_6_ [40,41].

## 2. Results and Discussion

### 2.1. Synthesis of the Rhodium (I) Complex

As a preliminary undertaking, the synthesis of 4-(diphenylphosphanyl)-phenyl]-*N*,*N*-dimethylmethanamine was initiated. The synthesis was adapted from the protocol reported by Togo and co-workers [42] and began with the amination of 4-bromobenzyl bromide by the addition of dimethyl amine in the presence of K_2_CO_3_. The bromine atom of 1-(4-bromophenyl)-*N*,*N*-dimethylmethanamine was substituted by a halogen–metal exchange with *n*-BuLi at −78 °C, following by a reaction with chlorodiphenylphosphine. Due to the practical constraints associated with the handling of the resultant phosphine, its oxidation was carried out using H_2_O_2_. Subsequent to the purification process, the phosphine was reduced with an excess of trichlorosilane. The phosphorus ligand was obtained in three steps, resulting in an overyield of 63%.

**Scheme 2:**
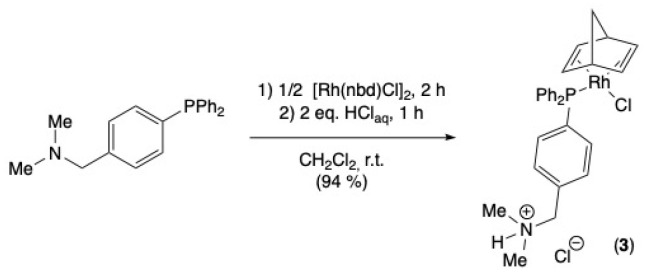
Synthesis of cationic [RhCl(**2**)(nbd)] complex (**3**).

The chloro-*P*-{[4-(diphenylphosphanyl)phenyl]-*N*,*N*-dimethylmethanammonio} (norbornadiene)rhodium(I) chloride complex (**3**) was obtained by reacting the phosphine previously obtained and the [RhCl(nbd)]_2_ complex (nbd = bicyclo[2.2.1]hepta-2,5-diene) in CH_2_Cl_2_. The cationic complex **3** was subsequently obtained by protonation with HCl and isolated by precipitation with a yield of 94% (Scheme 2). The coordination of the metal center was confirmed through the use of ^31^P{^1^H} NMR spectroscopy, which displayed a substantial weak-field shift of the phosphorus atom following its coordination with the rhodium precursor (^31^P{^1^H} NMR chemical shifts of −6.0 and 31.3 ppm for free and coordinated phosphine, respectively). Furthermore, the signal associated with complex **3** is consistent with a doublet that exhibits a coupling constant indicative of rhodium complexes (^1^*J*_RhP_ = 170.7 Hz) [43]. The ^1^H NMR spectrum revealed the presence of a signal in the weak fields at 12.53 ppm, indicative of the acid proton attributed to the amine, thereby confirming its protonation.

### 2.2. Formation of the Inclusion Complex

**Figure 2:**
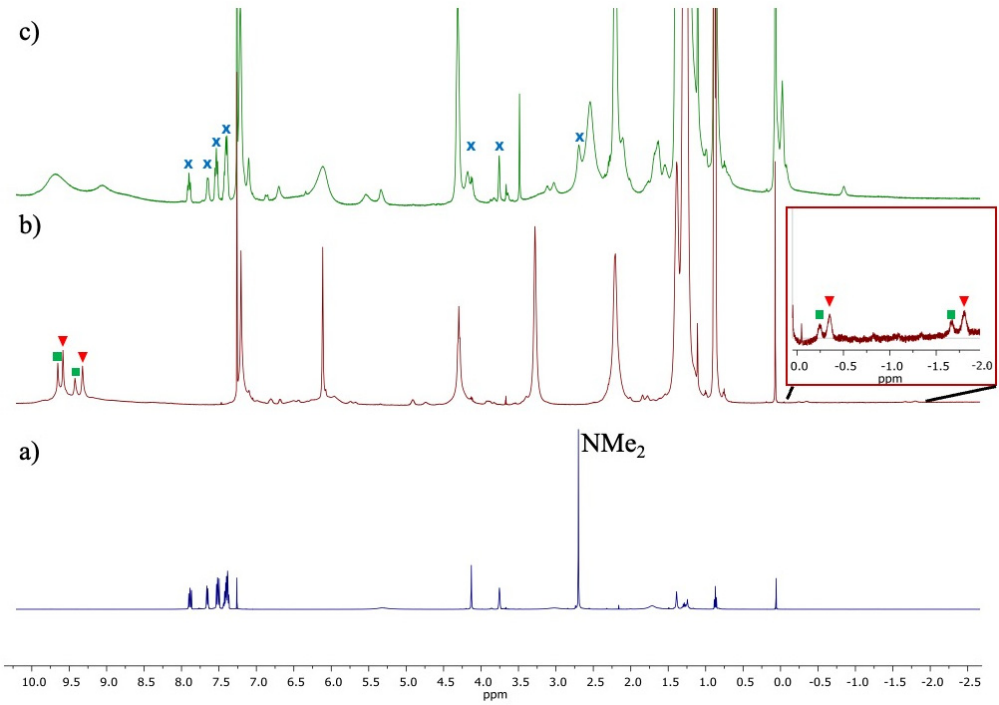
^1^H NMR spectra of the following: free complex **3** (**a**); encapsulated complex **3** (**3**⊂**1**_6_; with **3**⊂**1**_6_(8-H_2_O) red triangle and **3**⊂**1**_6_(15-H_2_O) green square) (**b**); after addition of NEt_4_Cl to **3**⊂**1**_6_ with signals from free **3** marked **X** (**c**).

The encapsulation of cationic complex **3** was studied in the presence of **1**_6_ ([1] = 26 mM and [3] = 4 mM) in H_2_O-saturated CDCl_3_. Following a 30 min stirring period, the formation of the inclusion complex **3**⊂**1**_6_ was observed by ^1^H NMR spectroscopy (Figure 2). Indeed, the appearance of signals in the negative zone (at −0.29 and −1.75 ppm), attributed to the methyl groups of the amine, was observed, synonymous with a species interacting with the internal walls of the cavity of **1**_6_. In addition to the substantial observed shielding (*δ* = 2.96 ppm in the “free” complex **3**), desymmetrization and signal splitting were also observed. The loss of symmetry can be attributed to the distinct electronic environment in which each methyl was localized. With regard to the signal splitting, the observed intensities of the peaks appear to be in direct correspondence with the intensities of the phenolic groups of **1**_6_(8-H_2_O) and **1**_6_(15-H_2_O) [44]. This observation suggests the encapsulation of **3** within these two capsules. The addition of NEt_4_Cl (5 equivalents), a competitor to encapsulation, to the solution of the inclusion complex **3**⊂**1**_6_ resulted in the release of the rhodium(I) complex **3**.

**Figure 3:**
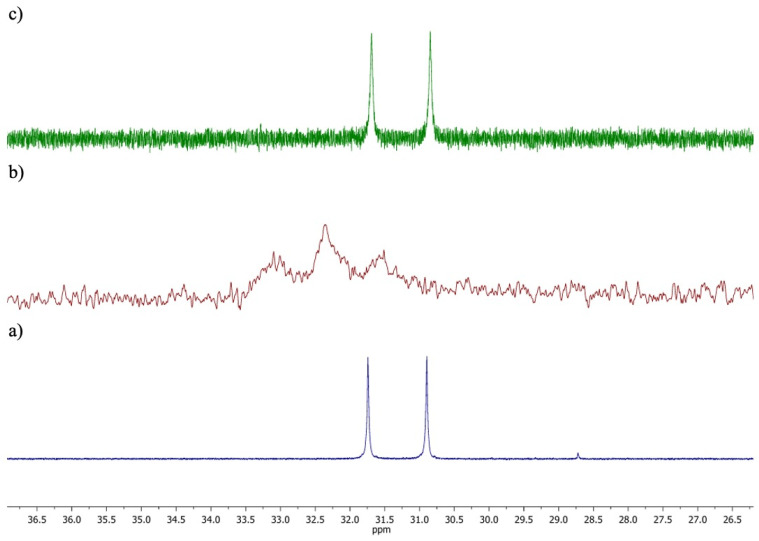
^1^P{^1^H} NMR spectra of the following: free complex **3** (**a**); encapsulated complex **3** (**3**⊂**1**_6_) (**b**); after addition of NEt_4_Cl to **3**⊂**1**_6_ (**c**).

The transition to a novel electronic environment was demonstrated to induce a modification of the ^31^P{^1^H} chemical shift. The initial doublet of **3** is divided into two tangled large doublets in the presence of **1**_6_. The observed signals can be attributed to the two inclusion complexes generated with capsules **1**_6_(8-H_2_O) and **1**_6_(15-H_2_O). The incorporation of five equivalents of NEt_4_Cl was demonstrated to induce the regeneration of the original spectrum, as previously observed (Figure 3).

**Figure 4:**
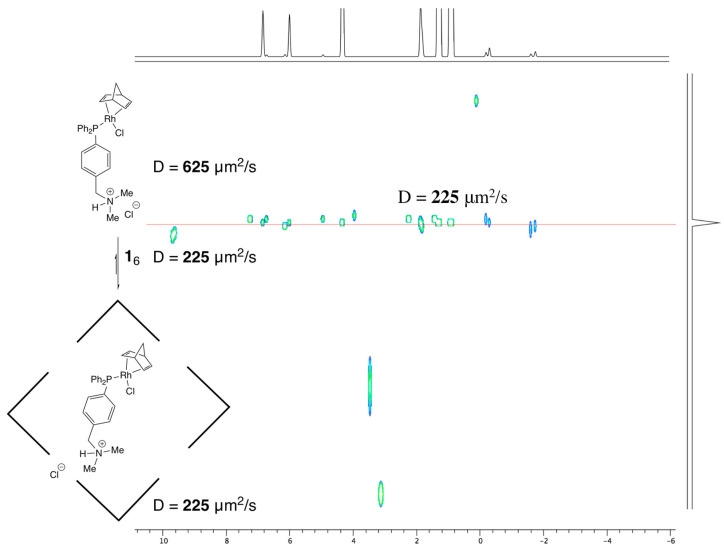
DOSY NMR of inclusion complex **3**⊂**1**_6_.

Additionally, signals in the negative part of the ^1^H NMR spectrum were also observed in the DOSY NMR experiment. The analysis revealed the presence of a single species attributed to the inclusion complex **3**⊂**1**_6_ (diffusion coefficient 225 μm^2^s^−1^; hydrodynamic volume 18,800 Å^3^; Figure 4). This value was found to be equivalent to that of capsule **1**_6_ (225 μm^2^s^−1^; hydrodynamic volume 18,800 Å^3^) and distinctly different from that of “free” complex **3** (diffusion coefficient 625 μm^2^s^−1^; hydrodynamic volume 877 Å^3^). It was determined that the presence of capsule **1**_6_ results in the absence of signals corresponding to “free” complex **3**. Given the finding that the values of hydrodynamic volumes **1**_6_ and **3**⊂**1**_6_ were identical, it could be deduced that the encapsulation of **3**, with an estimated volume of 610 Å^3^ and an occupancy factor of the inner cavity of 44%, close to the ideal value of 55 ± 9% [45], did not result in substantial modification of the structure of the supramolecular capsule.

**Figure 5:**
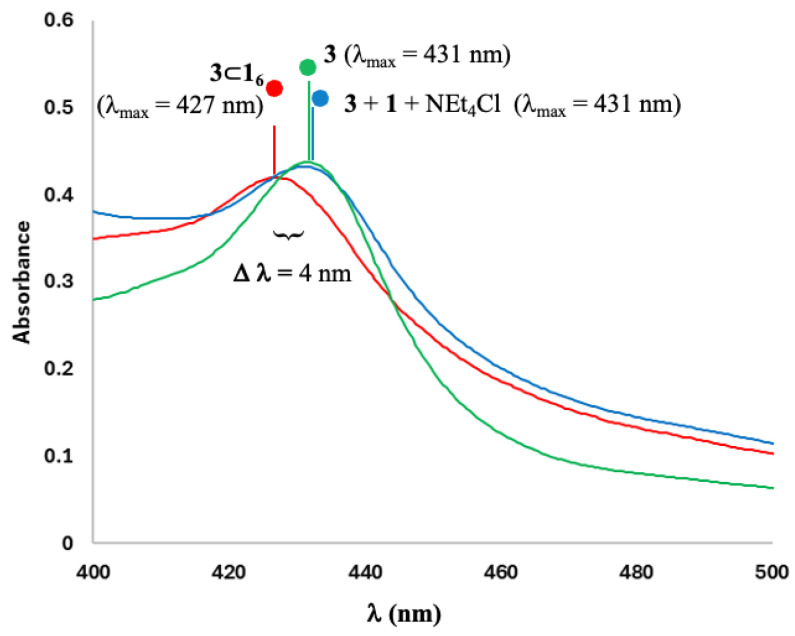
UV/Vis measurements of free complex **3** (in green with *λ*_max_ = 431 nm), encapsulated complex **3** (**3**⊂**1**_6_; in red with *λ*_max_ = 427 nm) and after addition of NEt_4_Cl to **3**⊂**1**_6_ (in blue with *λ*_max_ = 431 nm).

The formation of the inclusion complex was subsequently confirmed through the utilization of UV-visible spectroscopy (Figure 5). The maximum of the extinction coefficient of complex **3** (*λ* = 431 nm) underwent a weak bathochromic effect of 4 nm by the addition of **1**_6_ (*λ* = 427 nm), in agreement with the formation of an encapsulation phenomenon (**3**⊂**1**_6_) [23,24,25,40]. The release of **3** in solution by the addition of NEt_4_Cl was also confirmed by an absorbance maximum corresponding to the free complex (*λ* = 431 nm), once again demonstrating the reversibility of the encapsulation of complex **3** in the hexameric capsule **1**_6_.

### 2.3. Rhodium-Catalyzed [2+2+2] Cycloaddition

**Scheme 3:**
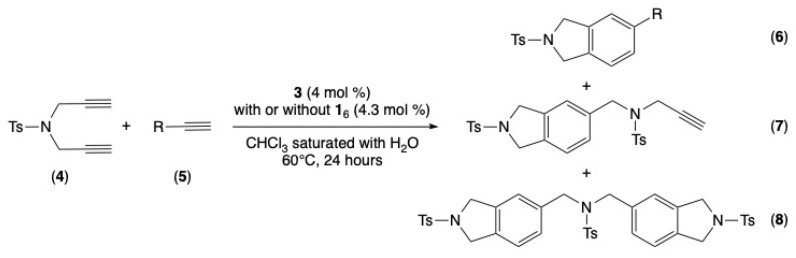
Rhodium-catalyzed [2+2+2] cycloaddition of *N*,*N*-dipropargyl-*p*-toluenesulfonamide **4** with arylacetylene **5**.

The well-documented reactivity of rhodium(I) complexes with [2+2+2] cycloaddition prompted the investigation of the inclusion complex **3**⊂**1**_6_ as a catalyst for this reaction. The model reaction studied is the [2+2+2] cycloaddition of *N*,*N*-dipropargyl-*p*-toluenesulfonamide **4** with phenylacetylene **5a**. Consequently, this reaction yields three distinct products, each corresponding to a specific chemical outcome. The first product, designated as **6a**, is a heterocycloaddition product, while the second product, labeled **7**, is a homocycloaddition product. The third product, designated as **8**, is a bishomocycloaddition adduct, which is formed through the reaction of **4** and **7**. To investigate the distribution of the products, conditions to achieve full conversion were used, with 100 µmol of **4**, an excess of **5a** and a catalytic loading of **3**⊂**1**_6_ of 4 mol % in H_2_O-saturated CHCl_3_ at 60 °C for 24 h (Scheme 3).

Complex **3** was initially evaluated in the presence of two equivalents of phenylacetylene **5a**, and its activity was observed, irrespective of the presence of capsule **1**_6_ (Table 1, entries 1 and 2). Full conversions could be observed, with heterocycloaddition product formation of 44% and 62% for **3** and **3**⊂**1**_6_, respectively. In the absence of the rhodium complex, no reactivity was observed, with or without capsule **1**_6_ (Table 1, entries 3 and 4). Furthermore, the addition of a competitor to the encapsulation, corresponding to NEt_4_Cl (20 mol %) to **3**⊂**1**_6_, resulted in a product distribution intermediate to those observed with **3** and **3**⊂**1**_6_ as precatalysts (Table 1, entry 5). The observed phenomenon could be attributed to the incomplete release of the catalytic species into the solution, which remained entrapped within capsule **1**_6_. Slight discrepancies in the distribution of products resulting from a test conducted with D_2_O-saturated solvent as opposed to H_2_O, with no deuterium incorporation into the products (Table 1, entry 6), imply that the capsule should not affect the conventional mechanism for this reaction (see Scheme 1). The insertion of the carbon–carbon triple bond into an Rh-H intermediate is unequivocally ruled out. It is noteworthy that in the presence of methanol (25% *v*/*v*) in the reaction medium, the distribution of catalysis products remains unaltered by the presence of capsule **7**_6_ (Table 1, entries 7 and 8). This outcome can be attributed to the disruption of the hydrogen bonding network, likely partially due to the amount of MeOH utilized, which results in the opening of the capsule [46]. These tests demonstrated that the presence of the capsule promoted the formation of the product of interest, **6a**, by enabling cycloaddition to take place inside the cavity formed by **1**_6_.

**Figure 6:**
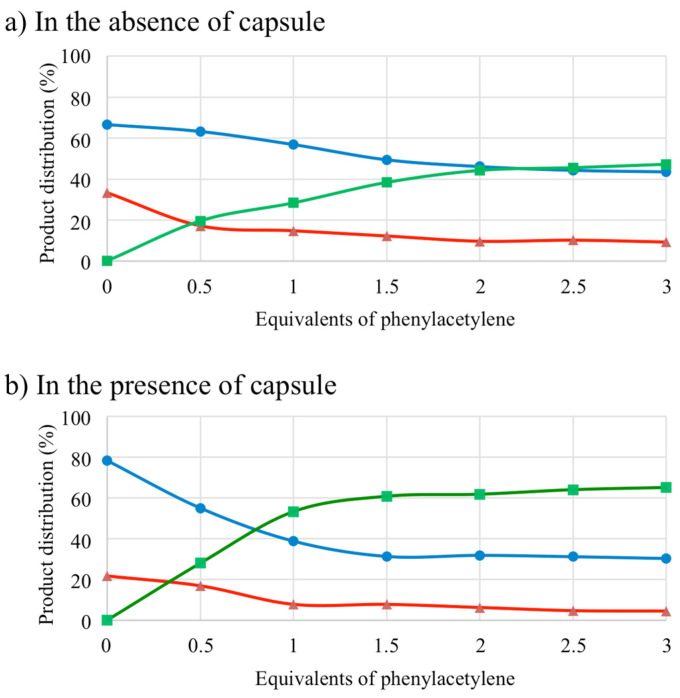
Rhodium-catalyzed [2+2+2] cycloaddition of N,N-dipropargyl-p-toluenesulfonamide **4** with different proportions of phenylacetylene **5a**, formation of heterocycloaddition product **6a** (in green) and homocycloaddition products **7** (in blue) and **8** (in red).

Subsequently, an investigation was conducted into the impact of the phenylacetylene **5a** equivalent number on this reaction. Consequently, the unencapsulated complex **3** was initially assessed (Figure 6a). The predominant formation of homocycloaddition product **7** was observed for stoichiometry below two equivalents of **5a**. At the critical value of two equivalents of **5a**, a similar formation of product **7** and the product of interest, **6a**, was obtained (46% and 44%, respectively). At higher stoichiometries, the **6a** product exhibited a marginal predominance. The bishomocycloaddition product **8** was observed in the minority under different conditions (for one equivalent of **5a**: 15% formation of **8**, for three equivalents of **5a**: 9% formation of **8**).

In a subsequent step, the incorporation of capsule **1**_6_ served to reverse the selectivity of this reaction (Figure 6b). In accordance with the experimental findings, the heterocycloaddition product **6a** was predominantly formed from one equivalent of **5a**. The formation of 53% of **6a** and 39% of **7** was measured. This trend remained constant for a higher number of **5a** equivalents. As anticipated, the formation of bishomocycloaddition product **8** was diminished in the presence of **1**_6_ (for one equivalent of **5a**: 8% formation of **8**, for three equivalents of **5a**: 5% formation of **8**). The hypothesis that this product was formed in hexameric capsule **1**_6_ was not supported by the size of the reagents involved. The decomposition of the hexameric capsule into a pentameric capsular species [47], i.e., a more accessible catalytic active species, or a minor release of rhodium complex into solution, could provide a potential explanation for the formation of **8**. The increase in the product of interest, **6a**, when the capsule **1**_6_ was added may be explained by the difference in size of the reagents, which favored diffusion of the less sterically hindered alkyne. Furthermore, the varying degrees of affinity exhibited by the reagents for the cavity may be a subject of debate. It was evident that 1,6-diyne **4** exhibited a higher degree of reactivity in comparison to phenylacetylene **5a**. The heightened reactivity of **4** resulted in the predominance of homocycloaddition product **7** under stoichiometric conditions, particularly when the catalyst was “free” in solution. The incorporation of the capsule **1**_6_ modified the diffusion of the substrates toward the metal, thereby affecting the reactivity of the alkynes with the catalytic species. Consequently, the predominant formation of the heterocycloaddition product **6** was observed. It is noteworthy that the homocycloaddition product [2+2+2] of phenylacetylene **5a** was only formed in trace amounts. No oligomerization products were detected.

It was demonstrated that the presence of the supramolecular capsule **1**_6_ favors the formation of the heterocycloaddition product **6**. Consequently, a series of thirteen alkynes were subjected to testing. The alkynes included in the study were phenylacetylene (**5a**), 4-ethynyltoluene (**5b**), 4-ethynylanisole (**5c**), 1-*tert*-butyl-4-ethynylbenzene (**5d**), 1-chloro-4-ethynylbenzene (**5e**), 1-fluoro-4-ethynylbenzene (**5f**), 2-ethynyltoluene (**5g**), 1-fluoro-2-ethynylbenzene (**5h**), 2-ethynylnaphthalene (**5i**), 2-ethynyl-6-methoxynaphthalene (**5j**), 9-ethynylphenanthrene (**5k**), ethynyltrimethylsilane (**5l**) and propargyl alcohol (**5m**). Catalytic tests were carried out with two equivalents of alkylene **5a–l**, corresponding to the stoichiometry, in the case of phenylacetylene **5a**, for which in the absence of **1**_6_, the formation of **6a** and **7** became similar (Table 2).

The employment of the inclusion complex **3**⊂**1**_6_ for the series of arylacetylenes **5a–k** resulted in an enhancement of the heterocycloaddition products **6a–k**, which became the predominant product for all the distinct tested alkynes. In this series, the increase in product **6** varied by a factor of 1.2 to 1.7 times greater compared with the “free” catalytic system. The most significant increase was observed in the case of 2-ethynyltoluene **5g**, where the methyl substituent was positioned in close proximity to the alkyne (**6g** formation of 32% and 54% with, respectively, **3** and **3**⊂**1**_6_; Table 2, entries 13 and 14). For catalysis by unencapsulated **3**, this proximity resulted in a reduction of alkyne reactivity due to steric hindrance induced by the methyl group. This phenomenon was less evident when **1**_6_ was present, attributable to the close proximity of reagents **4** and **5g** induced by the cavity of **1**_6_. A comparable effect was observed for 1-fluoro-2-ethynylbenzene **5h** (**6h** formation of 37% and 59% with, respectively, **3** and **1**_6_; Table 2, entries 15 and 16). The presence of a bulky substituent, as with 1-*tert*-butyl-4-ethynylbenzene **5d**, which hindered the diffusion of the substrate into the cavity of **1**_6_, did not inhibit the formation of the product **6e** (**6e** formation of 38% and 58% with, respectively, **3** and **3**⊂**1**_6_; Table 2, entries 7 and 8). In the case of extended aromatic systems **5i–k**, the formation of products **6i–k** with the “free” precatalyst **3** was predominant, with the exception of 9-ethynylphenanthrene **5k**. Nevertheless, it was observed that the incorporation of **1**_6_ led to an enhancement in the formation of products **6i–k** (Table 2, entries 17–22).

However, it should be noted that this system was not without limitations. For instance, in the case of ethynyltrimethylsilane (**5l**), the selectivity toward heterocycloaddition product **6l** diminished, and homocycloaddition product **7** was formed in the majority of cases, whether in the presence (46%) or absence (47%) of the capsule. It was noteworthy that this substrate exhibited a higher quantity (12%) of bishomocycloaddition product **8** (Table 2, entries 23 and 24). This observation indicated that substrate **5l** could potentially contribute to the destabilization of the inclusion complex **3**⊂**1**_6_, which could result in the release of the catalytic center or the opening of the capsule leading to the formation of a pentameric structure. Consequently, these may facilitate the formation of the sterically hindered product **8**. It has been observed that the utilization of propargyl alcohol (**5m**) as a substrate results in a modification of the 60-hydrogen bond network of the capsule **7**_6_. It was observed that, in the presence or absence of the supramolecular capsule **7**_6_, the heterocycloaddition product **6m** was formed in a proportion of 85% with the latter reagent (Table 2, entries 25 and 26).

## 3. Materials and Methods

### 3.1. General

All the manipulations were carried out under dry argon. Routine ^1^H, ^13^C{^1^H}, ^31^P{^1^H} and DOSY spectra were recorded with Bruker FT instruments (AC 300, 500 and 600). CDCl_3_ was degassed, passed down a 5 cm thick basic alumina column and stored under argon. NMR spectra were calibrated according to residual protonated solvent (*δ* = 7.26 ppm and 77.16 ppm for ^1^H and ^13^C{^1^H} NMR, respectively). ^31^P{^1^H} NMR spectroscopic data are given relative to external H_3_PO_4_. Chemical shifts and coupling constants are reported in ppm and Hz, respectively.

UV-visible spectra were recorded on an Agilent Technologies Cary 60 UV-Vis spectrometer. Mass spectra were recorded on a Bruker MicroTOF spectrometer (ESI-TOF).

4-Bromobenzyl bromide, dimethylamine, tetraethylammonium chloride, [RhCl(nbd)]_2_, phenylacetylene, 4-ethynyltoluene, 2-ethynyltoluene, 4-ethynylanisole, 4-*tert*-butylphenylacetylene, 1-chloro-4-ethynylbenzene, 1-fluoro-4-ethynylbenzene, 1-fluoro-2-ethynylbenzene, 2-ethynyl-naphthalene, 2-ethynyl-6-methoxynaphthalene, 9-ethynylphenanthrene, ethynyltrimethylsilane and propargyl alcohol are commercially available products (Alfa Aesar, Sigma-Aldrich or Abcr) and were used as received without any further purification. 2,8,14,20-Tetra-undecyl-resorcin[4]arene (**1**) [48], 4-(diphenylphosphanyl)-phenyl]-*N*,*N*-dimethylmethanamine [42] and *N*,*N*-dipropargyl-*p*-toluenesulfonamide (**4**) [49] were prepared by following the literature procedures.

### 3.2. Synthesis of Chloro-P-{[4-(Diphenylphosphanyl)phenyl]-N,N-dimethylmethanammonio} (Norbornadiene)rhodium(I) Chloride (***3***)

The 1-(4-(diphenylphosphaneyl)phenyl)-*N*,*N*-dimehylmethanamine (227 mg, 0.711 mmol) and [RhCl(nbd)]_2_ (163 mg, 0.354 mmol) were dissolved in CH_2_Cl_2_ (10 mL) and the resulting solution was stirred at room temperature. After 2 h, HCl (37% in water, 0.118 mL, 1.423 mmol) was added and the reaction mixture was stirred for 1 h before the addition of MgSO_4_, which was eliminated by filtration. The filtrate was concentrated to ca. 2 mL and *n*-hexane (40 mL) was added. The precipitate was filtered, washed with *n*-hexane (3 × 8 mL) and dried under vacuum, yielding a brown solid (392 mg, 94%). ^1^H NMR (500 MHz, CDCl_3_): *δ* = 12.53 (s, 1H, N*H*(CH_3_)_2_), 7.92–7.88 (m, 2H, arom CH of P(C_6_H_4_)), 7.66 (d, 2H, arom CH of P(C_6_H_4_), ^3^*J*_HH_ = 8.5 Hz), 7.54–7.50 (m, 4H, arom CH of P(C_6_H_5_)_2_), 7.44–7.38 (m, 6H, arom CH of P(C_6_H_5_)_2_), 5.32 (brs, 2H, CH of nbd), 4.13 (d, 2H, NCH_2_, ^3^*J*_HH_ = 5.0 Hz), 3.76 (s, 2H, CH of nbd), 3.02 (brs, 2H, CH of nbd), 2.71 (d, 6H, NH(C*H*_3_)_2_, ^3^*J*_HH_ = 5.0 Hz), 1.39 (s, 2H, CH_2_ of nbd); ^13^C{^1^H} NMR (126 MHz, CDCl_3_): *δ* = 135.84 (d, arom CH of P(C_6_H_4_), ^2^*J*_CP_ = 12.2 Hz), 134.59 (d, arom C_quat_ of P(C_6_H_4_), ^1^*J*_CP_ = 38.8 Hz), 134.19 (d, arom CH of P(C_6_H_5_), ^2^*J*_CP_ = 11.7 Hz), 130.93 (d, arom CH of P(C_6_H_4_), ^3^*J*_CP_ = 10.2 Hz), 130.87 (d, arom CH of P(C_6_H_4_), ^4^*J*_CP_ = 2.5 Hz), 130.64 (d, arom C_quat_ of P(C_6_H_5_)_2_, ^1^*J*_CP_ = 42.7 Hz), 130.64 (d, arom CH of P(C_6_H_5_)_2_, ^4^*J*_CP_ = 2.3 Hz), 128.70 (d, arom CH of P(C_6_H_5_)_2_, ^3^*J*_CP_ = 10.0 Hz), 85.69 (brs, CH of nbd, CH=CH), 64.03 (d, CH_2_ of nbd, ^4^*J*_CP_ = 5.4 Hz), 60.87 (s, NCH_2_), 51.85 (brs, CH of nbd, CH=CH), 50.79 (d, CH of nbd, ^3^*J*_CP_ = 1.5 Hz), 42.61 (s, NH(CH_3_)_2_); ^31^P{^1^H} NMR (202 MHz, CDCl_3_): *δ* = 31.3 (d, PPh_2_, ^1^*J*_PRh_ = 170.7 Hz) ppm. MS (ESI-TOF): *m*/*z* = 550.09 [M − Cl]^+^, 514.12 [M − HCl − Cl]^+^ (expected isotopic profiles). Elemental analysis (%): calcd for C_28_H_31_NCl_2_PRh (586.34): C: 57.36; H: 5.33; N: 2.39; found C: 57.54; H: 5.51 N: 2.25.

### 3.3. General Procedure for the Rhodium-Catalyzed Cycloaddition

Under argon, the rhodium complex (**3**) (2.4 mg, 4 µmol, 4 mol %) was dissolved in H_2_O-saturated CHCl_3_ (1 mL). When necessary, resorcinarene (**1**) (28.7 mg, 26 µmol, 26 mol %) was added and the resulting solution was stirred at room temperature for 0.5 h. Then, *N*,*N*-dipropargyl-*p*-toluenesulfonamide (**4**; 24.7 mg, 100 µmol) and the corresponding amount of the desired alkyne were added. The reaction mixture was heated at 60 °C for 24 h. An aliquot of the solution was analyzed by ^1^H NMR. The products were unambiguously identified by ^1^H and ^13^C{^1^H} NMR after their isolation by flash chromatography on silica gel (eluent: petroleum ether/AcOEt: 7/3 *v*/*v*) (see Supplementary Materials).

## 4. Conclusions

In the present article, we reported on the use of a triphenylphosphine analogue, 1-(4-(diphenylphosphaneyl)phenyl)-*N*,*N*-dimethylmethanimine for intra-cavity catalytic application. Following coordination of the [RhCl(nbd)]_2_ and subsequent protonation of its amine function, this protonated phosphine demonstrated the capacity to stabilize the inclusion complex with the self-assembled capsule generated with six molecules of 2,8,14,20-tetra-undecyl-resorcin[4]arene and eight molecules of water. The formation of an inclusion complex was deduced from a combination of spectral measurements (UV-visible spectroscopy, ^1^H, ^31^P{^1^H} NMR and DOSY). The formation of the rhodium(I)-based inclusion complex was facilitated by π-cation interactions between the complex ligand and the aromatic inner rings of the cavity.

The inclusion complex was demonstrated to promote the formation of the heterocycloaddition product [2+2+2] between *N*,*N*-dipropargyl-*p*-toluenesulfonamide and arylacetylenic derivatives when compared with the “free” rhodium catalyst. In optimal conditions and with the utilization of merely two equivalents of arylacetylenes, the formation of these products of interest was found to be augmented by up to 69% in the case of 4-ethynylanisole, which corresponds to 1.4 times the amount observed with the “free” rhodium complex.

The employment of this readily accessible phosphine is expected to facilitate the immobilization of diverse organometallic complexes within supramolecular capsules comprising aromatic rings. Subsequent research will employ this strategy to examine the impact of confining catalytic centers within self-assembled capsules.

## Figures and Tables

**Table 1:** Control experiments ^1^.

Entry	3	Capsule (1_6_)	Additive	Conversion (%) ^2^	Product Distribution ^2^		
					6a (%)	7 (%)	8 (%)
1	Yes	No	/	100	44	46	10
2	Yes	Yes	/	100	62	32	6
3	No	No	/	0	0	0	0
4	No	Yes	/	0	0	0	0
5	Yes	Yes	NEt_4_Cl ^3^	100	53	40	7
6	Yes	Yes	D_2_O ^4^	100	65	30	5
7	Yes	No	MeOH ^5^	100	44	50	6
8	Yes	Yes	MeOH ^5^	100	43	50	7

^1^ Conditions: **4** (24.7 mg, 100 µmol), **5a** (22 μL, 200 µmol), H_2_O-saturated CHCl_3_ (1 mL), 60 °C, 24 h. If necessary: **3** (2.4 mg, 4 µmol, 4 mol %) and **1**_6_ (28.7 mg, 4.3 µmol, 4.3 mol %); ^2^ Determined by ^1^H NMR; ^3^ NEt_4_Cl (3.6 mg, 20 µmol, 20 mol %); ^4^ D_2_O instead of H_2_O; ^5^ MeOH (340 µL, 25% *v*/*v*).

**Table 2:** Rhodium-catalyzed [2+2+2] cycloaddition with different alkynes ^1^.

Entry	Alkyne		Capsule (1_6_)	Product Distribution ^2^		
				6 (%)	7 (%)	8 (%)
1	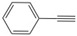	**5a**	No	44	46	10
2			Yes	62	32	6
3	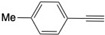	**5b**	No	39	50	11
4			Yes	57	37	6
5	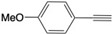	**5c**	No	48	41	11
6			Yes	69	28	3
7	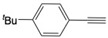	**5d**	No	38	50	12
8			Yes	58	36	6
9	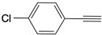	**5e**	No	47	45	8
10			Yes	60	34	6
11	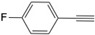	**5f**	No	40	49	11
12			Yes	60	34	6
13	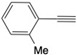	**5g**	No	32	54	14
14			Yes	54	37	9
15	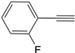	**5h**	No	37	51	12
16			Yes	59	32	9
17	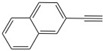	**5i**	No	52	40	8
18			Yes	63	31	6
19	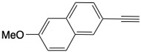	**5j**	No	48	43	9
20			Yes	63	32	6
21	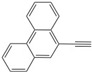	**5k**	No	43	46	11
22			Yes	53	38	9
23	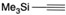	**5l**	No	40	50	10
24			Yes	41	47	12
25	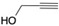	**5m**	No	85	15	0
26			Yes	85	15	0

^1^ Conditions: **4** (24.7 mg, 100 µmol), **5** (200 µmol), **3** (2.4 mg, 4 µmol, 4 mol %), H_2_O-saturated CHCl_3_ (1 mL), 60 °C, 24 h. If necessary: **1**_6_ (28.7 mg, 4.3 µmol, 4.3 mol %); ^2^ Determined by ^1^H NMR.

## Data Availability

The data presented in this study are available upon request from the corresponding author.

## Supplementary Materials

The following supporting information can be downloaded at: https://www.mdpi.com/article/10.3390/molecules30143052/s1, characterizing data of chloro-*P*-{[4-(diphenylphosphanyl) phenyl]-*N*,*N*-dimethylmethanammonio}(norbornadiene)rhodium(I) chloride (**3**) with Figure S1: ^1^H NMR spectrum, Figure S2: ^13^C{^1^H} NMR spectrum, Figure S3: ^31^P{1H} NMR spectrum, Figure S4: Mass spectrum (ESI-TOF) and Figure S5: DOSY NMR; characterizing data of 5-phenyl-2-tosylisoindoline (**6a**) with Figure S6: ^1^H NMR spectrum and Figure S7: ^13^C{^1^H} NMR spectrum; characterizing data of 5-(*p*-tolyl)-2-tosylisoindoline (**6b**) with Figure S8: ^1^H NMR spectrum and Figure S9: ^13^C{^1^H} NMR spectrum; characterizing data of 5-(4-methoxyphenyl)-2-tosylisoindoline (**6c**) with Figure S10: ^1^H NMR spectrum and Figure S11: ^13^C{^1^H} NMR spectrum; characterizing data of 5-(4-(*tert*-butyl)phenyl)-2-tosylisoindoline (**6d**) with Figure S12: ^1^H NMR spectrum and Figure S13: ^13^C{^1^H} NMR spectrum; characterizing data of 5-(4-chlorophenyl)-2-tosylisoindoline (**6e**) with Figure S14: ^1^H NMR spectrum and Figure S15: ^13^C{^1^H} NMR spectrum; characterizing data of 5-(4-fluorophenyl)-2-tosylisoindoline (**6f**) with Figure S16: ^1^H NMR spectrum, Figure S17: ^13^C{^1^H} NMR spectrum and Figure S18: ^19^F{^1^H} NMR spectrum; characterizing data of 5-(*o*-tolyl)-2-tosylisoindoline (**6g**) with Figure S19: ^1^H NMR spectrum and Figure S20: ^13^C{^1^H} NMR spectrum; characterizing data of 5-(2-fluorophenyl)-2-tosylisoindoline (**6h**) with Figure S21: Mass spectrum, Figure S22: ^1^H NMR spectrum, Figure S23: ^13^C{^1^H} NMR spectrum and Figure S24: ^19^F{^1^H} NMR spectrum; characterizing data of 5-(naphthalen-2-yl)-2-tosylisoindoline (**6i**) with Figure S25: Mass spectrum, Figure S26: ^1^H NMR spectrum and Figure S27: ^13^C{^1^H} NMR spectrum; characterizing data of 5-(6-methoxynaphthalen-2-yl)-2-tosylisoindoline (**6j**) with Figure S28: Mass spectrum, Figure S29: ^1^H NMR spectrum and Figure S30: ^13^C{^1^H} NMR spectrum; characterizing data of 5-(phenanthren-9-yl)-2-tosylisoindoline (**6k**) with Figure S31: Mass spectrum, Figure S32: ^1^H NMR spectrum and Figure S33: ^13^C{^1^H} NMR spectrum; characterizing data of 2-tosyl-5-(trimethylsilyl)isoindoline (**6l**) with Figure S34: Mass spectrum, Figure S35: ^1^H NMR spectrum and Figure S36: ^13^C{^1^H} NMR spectrum; characterizing data of (2-tosylisoindolin-5-yl)methanol (**6m**) with Figure S37: ^1^H NMR spectrum and Figure S38: ^13^C{^1^H} NMR spectrum; characterizing data of 4-methyl-*N*-(prop-2-yn-1-yl)-*N*-((2-tosylisoindolin-5-yl)methyl)benzenesulfonamide (**7**) with Figure S39: ^1^H NMR spectrum and Figure S40: ^13^C{^1^H} NMR spectrum; characterizing data of 4-methyl-*N*,*N*-bis((2-tosylisoindolin-5-yl)methyl)benzenesulfonamide (**8**) with Figure S41: ^1^H NMR spectrum and Figure S42: ^13^C{^1^H} NMR spectrum.
