# Supplementary material for: Homo- Versus Hetero- [2+2+2] Rhodium-Catalyzed Cycloaddition: Effect of a Self-Assembled Capsule on the Catalytic Outcome
Source: Molecules. 2025 Jul 21;30(14):3052. doi: 10.3390/molecules30143052 (PMC12300146; doi:10.3390/molecules30143052)
Supplement: Supplementary file 1 [file molecules-30-03052-s001.zip › molecules-3766850-supplementary.pdf]

Homo versus hetero [2+2+2] rhodium-catalyzed cycloaddition: effect of a self-assembled capsule on the catalytic outcome

Maxime Steinmetz and David Sémeril

Contents

Characterizing data of chloro-*P*-{[4-(diphenylphosphanyl) phenyl]-*N*,*N*-dimethylmethanammonio} (norbornadiene)rhodium(I) chloride (**3**) p 2

NMR description of the catalytic products

5-Phenyl-2-tosylisoindoline (**6a**) p 5

5-(*p*-Tolyl)-2-tosylisoindoline (**6b**) p 7

5-(4-Methoxyphenyl)-2-tosylisoindoline (**6c**) p 9

5-(4-(*tert*-Butyl)phenyl)-2-tosylisoindoline (**6d**) p 11

5-(4-Chlorophenyl)-2-tosylisoindoline (**6e**) p 13

5-(4-Fluorophenyl)-2-tosylisoindoline (**6f**) p 15

5-(*o*-Tolyl)-2-tosylisoindoline (**6g**) p 17

5-(2-Fluorophenyl)-2-tosylisoindoline (**6h**) p 19

5-(Naphthalen-2-yl)-2-tosylisoindoline (**6i**) p 22

5-(6-Methoxynaphthalen-2-yl)-2-tosylisoindoline (**6j**) p 24

5-(Phenanthren-9-yl)-2-tosylisoindoline (**6k**) p 26

2-Tosyl-5-(trimethylsilyl)isoindoline (**6l**) p 28

(2-Tosylisoindolin-5-yl)methanol (**6m**) p 30

4-Methyl-*N*-(prop-2-yn-1-yl)-*N*-((2-tosylisoindolin-5-yl)methyl) benzenesulfonamide (**7**) p 32

4-Methyl-*N*,*N*-bis((2-tosylisoindolin-5-yl)methyl)benzenesulfonamide (**8**) p 34

Chloro-*P*-{[4-(diphenylphosphanyl) phenyl]-*N*,*N*-dimethylmethanammonio}

(norbornadiene)rhodium(I) chloride (3)

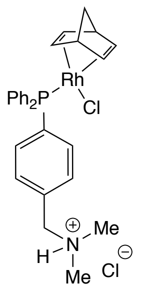

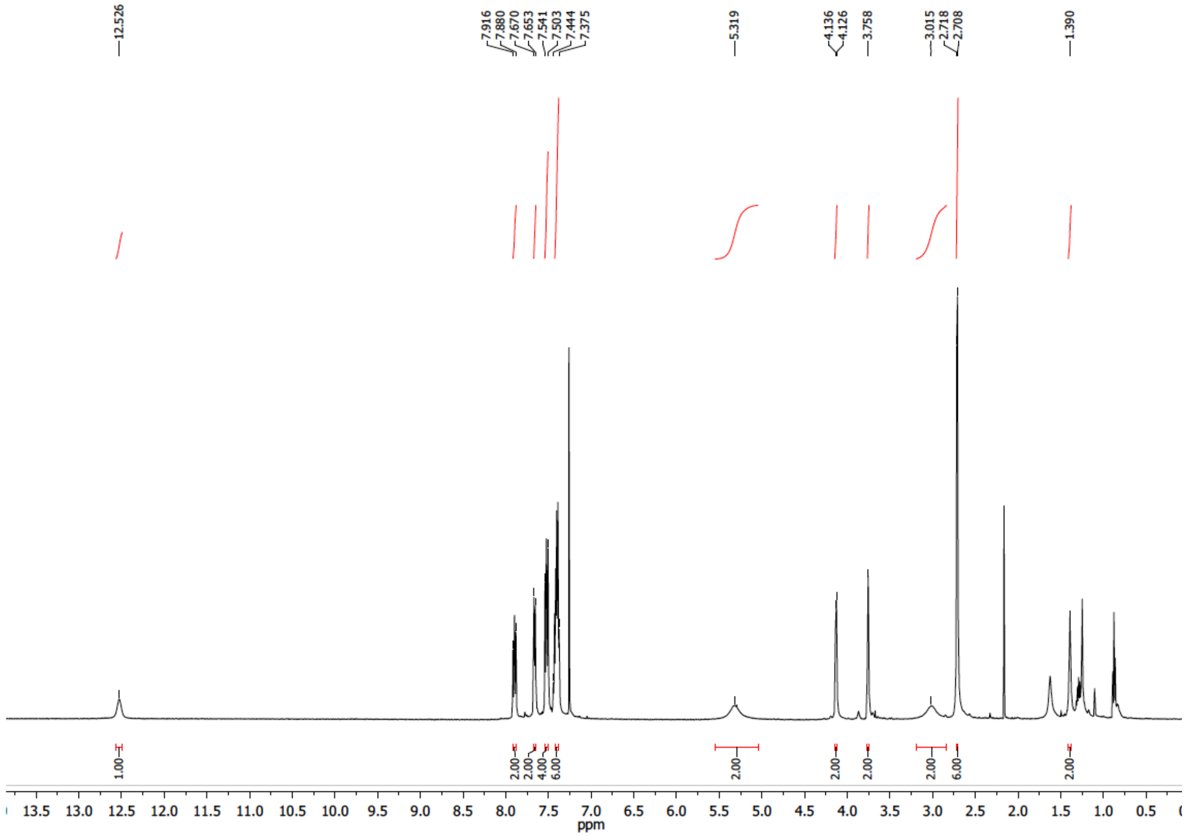

Figure S1. ^{1}H NMR spectrum ($CDCl_3$)

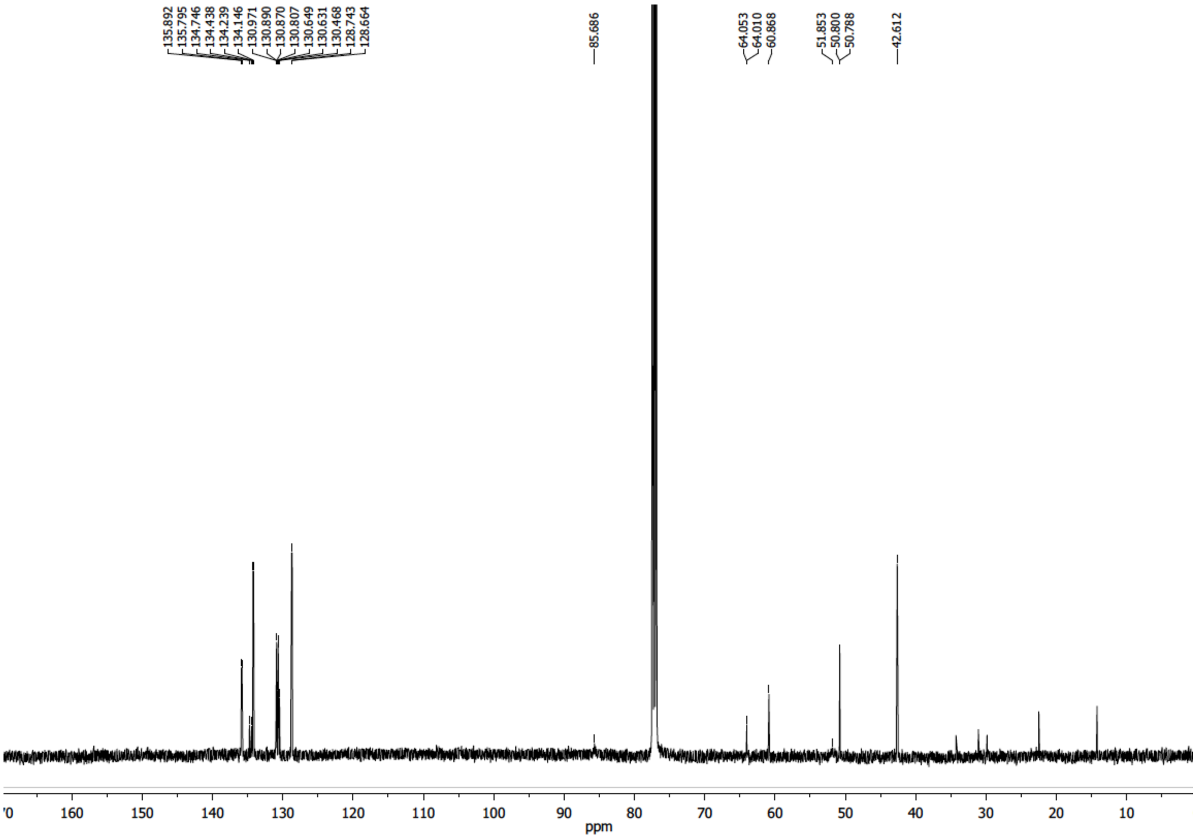

Figure S2. $^{13}C\{^{1}H\}$ NMR spectrum ($CDCl_3$)

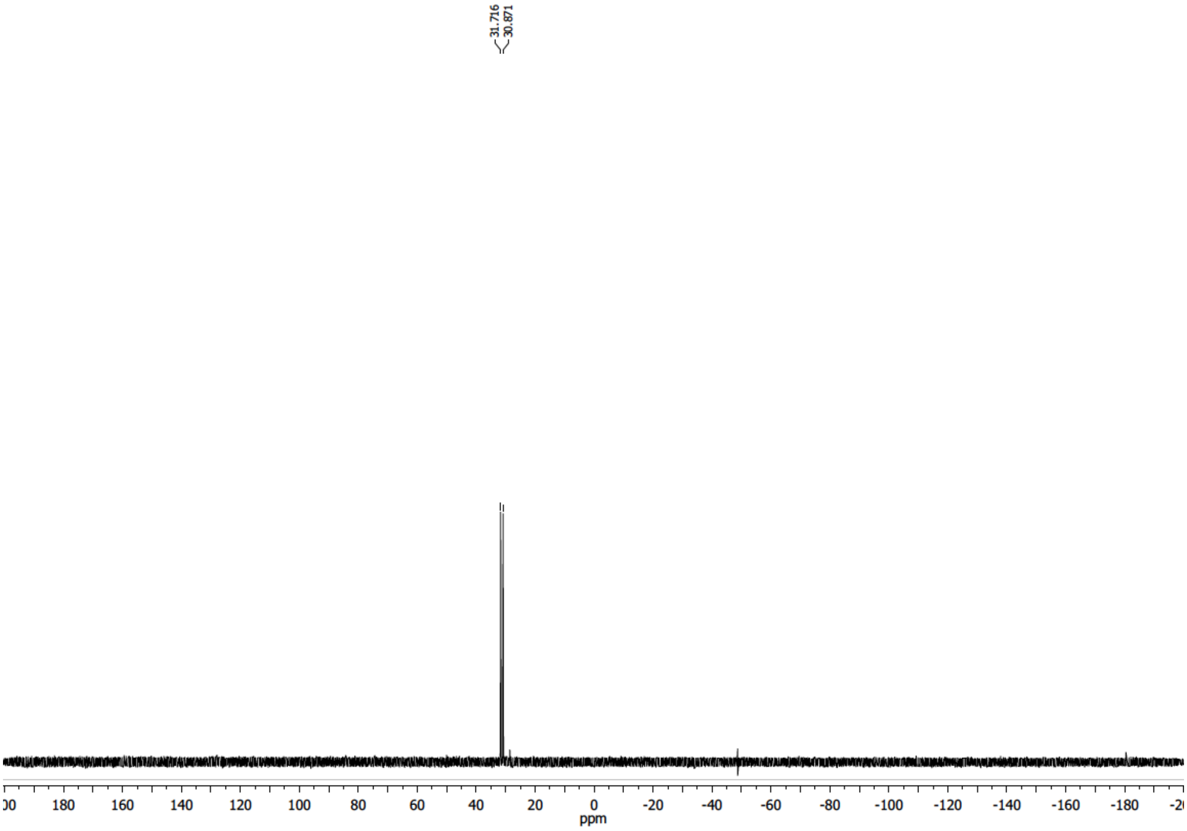

Figure S3. $^{31}P\{^{1}H\}$ NMR spectrum ($CDCl_3$)

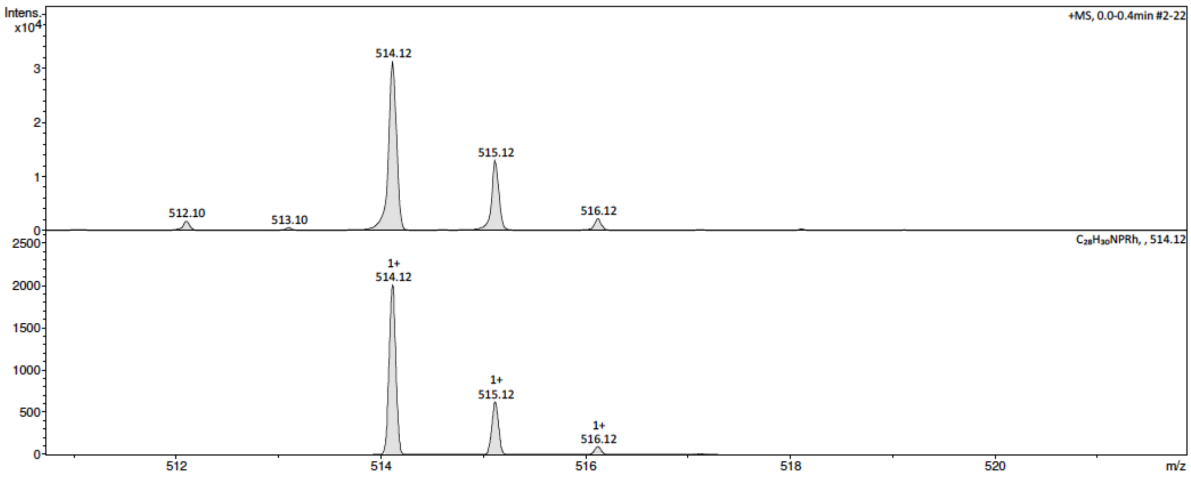

Figure S4. Mass spectrum (ESI-TOF): exp. spectrum (top); calc. spectrum (bottom) for $C_{28}H_{30}PNRh$ ($[M - HCl - Cl]^+$)

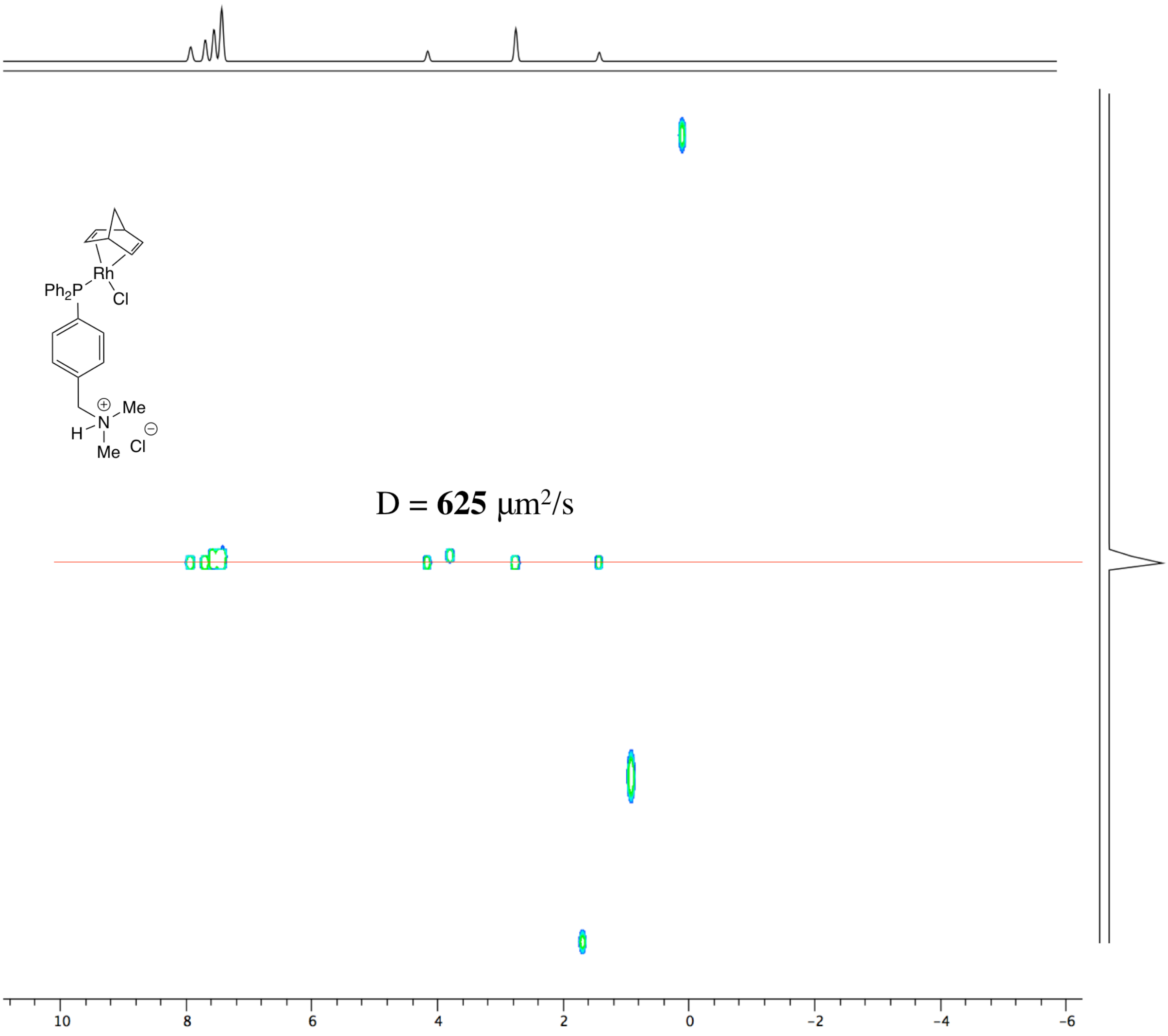

Figure S5. DOSY NMR of rhodium complex **3** (water saturated $CDCl_3$, 600 MHz, [**3**] = 20 mM).

5-Phenyl-2-tosylisoindoline (6a)

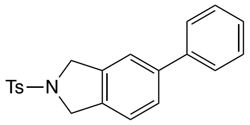

Rf: 0.57 petroleum ether/AcOEt: 7/3 v/v; isolated yield 57 % (20 mg). ^{1}H NMR (300 MHz, $CDCl_3$): δ = 7.79 (d, 2H, arom CH, $^3J_{HH}$ = 8.4 Hz), 7.53-7.31 (m, 9H, arom CH), 7.23 (d, 1H, arom CH, $^3J_{HH}$ = 8.1 Hz), 4.67 d, 2H, NCH_2, $^4J_{HH}$ = 2.7 Hz), 4.66 (d, 2H, NCH_2, $^4J_{HH}$ = 2.7 Hz), 2.41 (s, 3H, CH_3 of tosyl); $^{13}C\{^1H\}$ NMR (126 MHz, $CDCl_3$): δ = 143.86, 141.42, 140.70, 136.99, 135.28, 133.85 (6s, arom Cquat), 129.99, 128.99, 127.77, 127.69, 127.26, 127.06, 123.07, 121.47 (8s, arom CH), 53.87 (s, NCH_2), 53.69 (s, NCH_2), 21.66 (s, CH_3 of tosyl) ppm. Consistent with the literature data Z. Li, Y. Mao, Y. Sun, B. Ma, Y. Wang, G. Zhou, Y. Zhang, F. Zeng, Z. Wang, B. Li, *Results Chem*. **2024**, *7*, 101523.

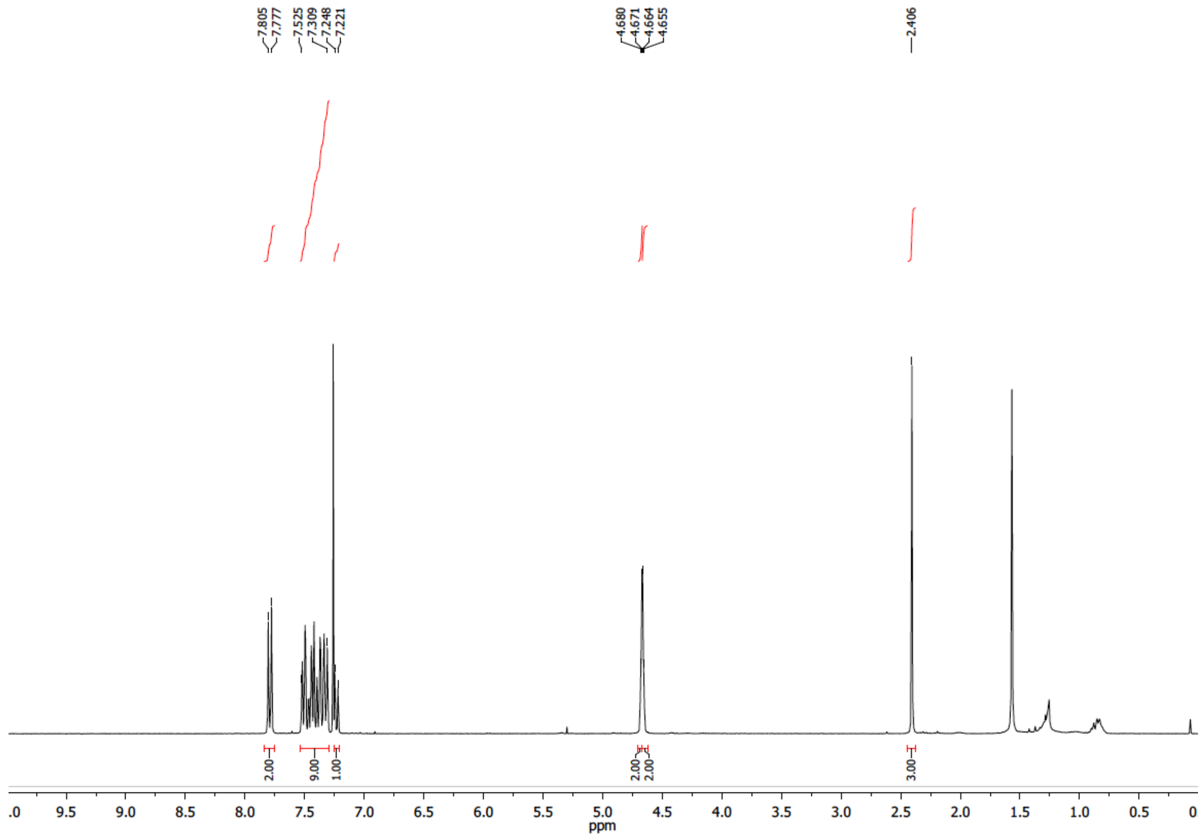

Figure S6. ^{1}H NMR spectrum ($CDCl_3$)

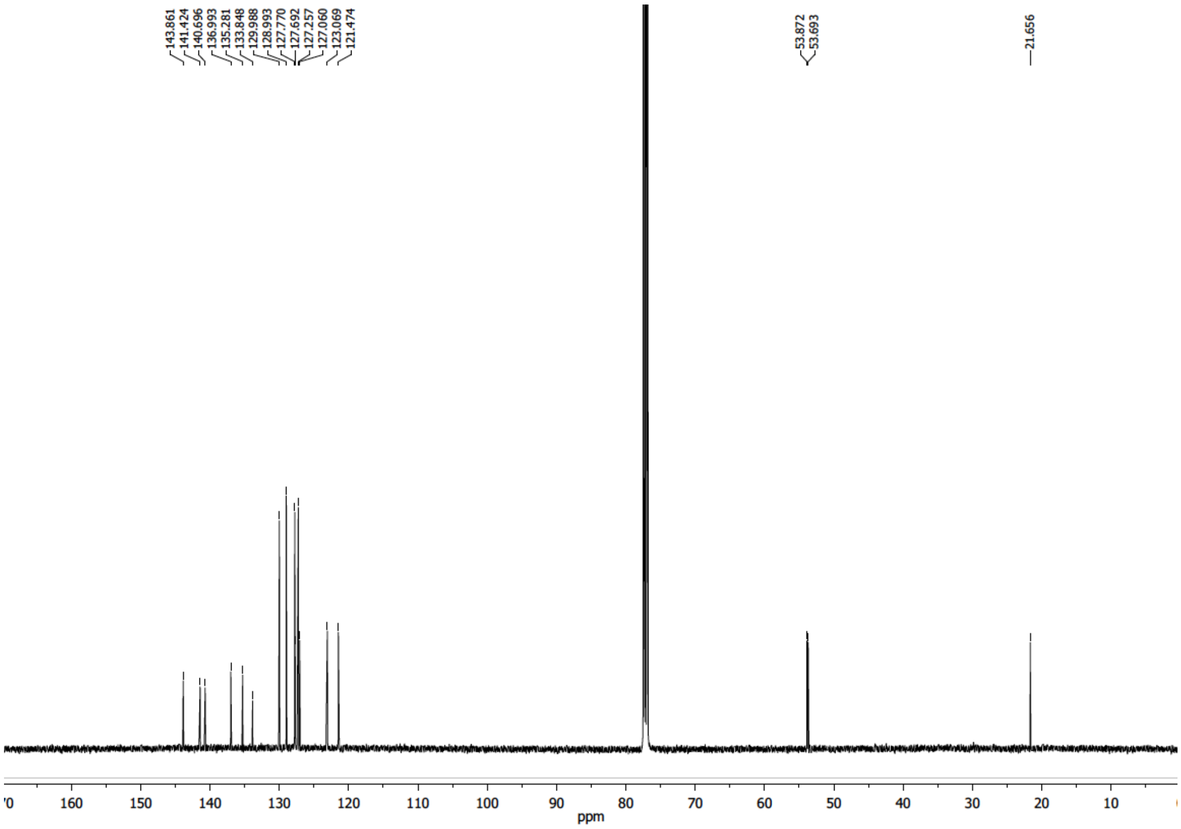

Figure S7. $^{13}C\{^1H\}$ NMR spectrum ($CDCl_3$)

5-(*p*-Tolyl)-2-tosylisoindoline (6b)

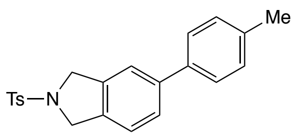

Rf: 0.79 petroleum ether/AcOEt: 7/3 v/v; isolated yield 53 % (19 mg). ^{1}H NMR (500 MHz, $CDCl_3$): δ = 7.79 (d, 2H, arom CH, $^{3}J_{HH}$ = 8.0 Hz), 7.43 (dd, 1H, arom CH, $^{3}J_{HH}$ = 8.0 Hz, $^{4}J_{HH}$ = 2.0 Hz), 7.40 (d, 2H, arom CH, $^{3}J_{HH}$ = 8.0 Hz), 7.35 (s, 1H, arom CH), 7.32 (d, 2H, arom CH, $^{3}J_{HH}$ = 8.0 Hz), 7.23-7.20 (m, 3H, arom CH), 4.66 (d, 2H, NCH_2, $^{4}J_{HH}$ = 2.5 Hz), 4.65 (d, 2H, CH_2, $^{4}J_{HH}$ = 1.5 Hz), 2.40 (s, 3H, CH_3 of tosyl), 2.38 (s, 3H, CH_3); $^{13}C\{^{1}H\}$ NMR (126 MHz, $CDCl_3$): δ = 143.84, 141.35, 137.81, 137.56, 136.95, 134.98, 133.87 (7s, arom Cquat), 129.98, 129.71, 127.78, 127.08, 126.86, 123.02, 121.25 (7s, arom CH), 53.89 (s, NCH_2), 53.71 (s, NCH_2), 21.66 (s, CH_3 of tosyl), 21.23 (s, CH_3) ppm. Consistent with the literature data Z. Li, Y. Mao, Y. Sun, B. Ma, Y. Wang, G. Zhou, Y. Zhang, F. Zeng, Z. Wang, B. Li, *Results Chem*. **2024**, *7*, 101523.

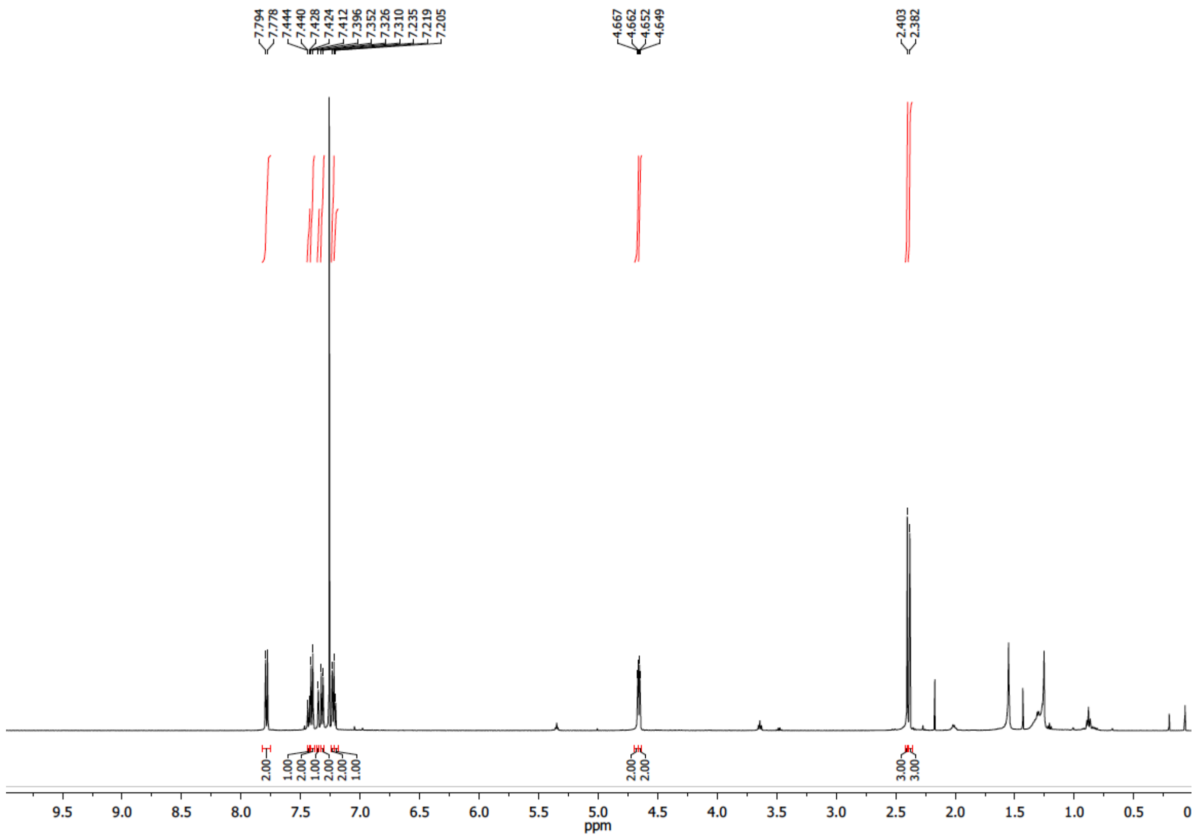

Figure S8. ^{1}H NMR spectrum ($CDCl_3$)

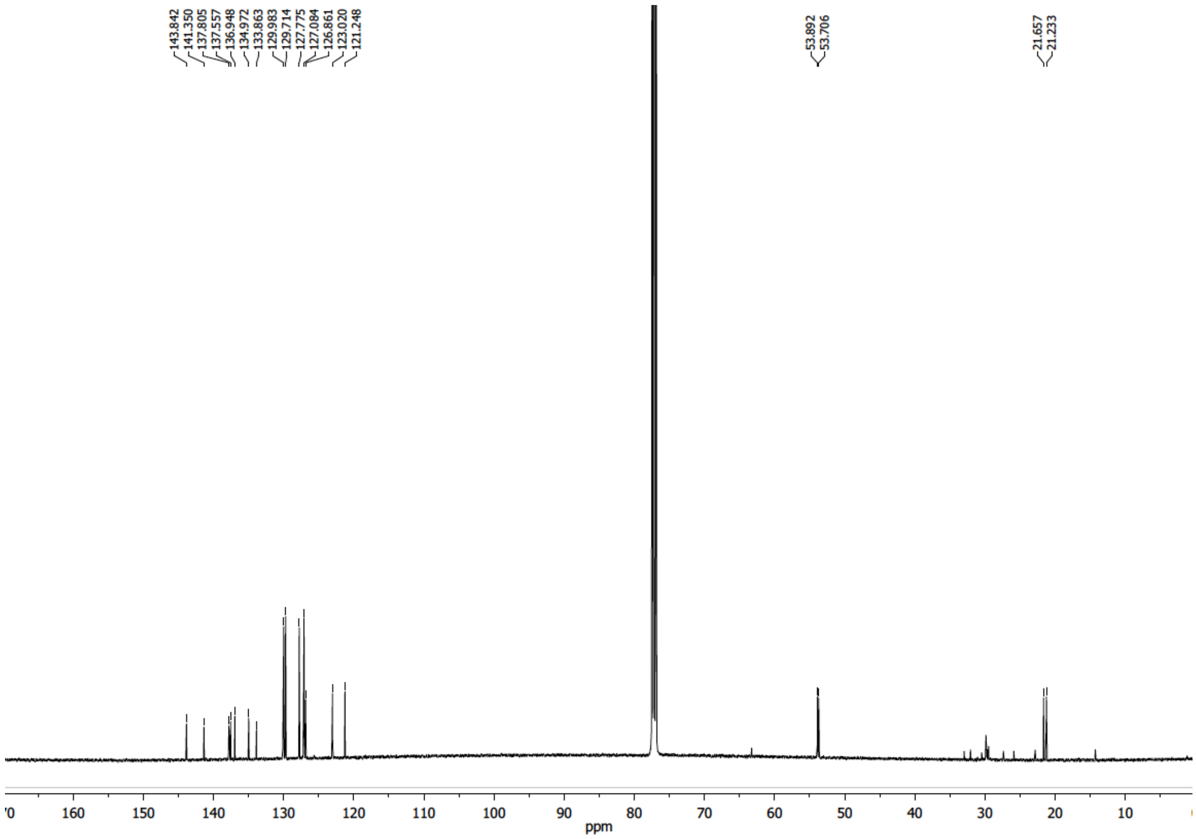

Figure S9. $^{13}C\{^1H\}$ NMR spectrum ($CDCl_3$)

5-(4-Methoxyphenyl)-2-tosylisoindoline (6c)

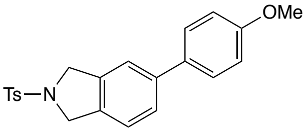

Rf: 0.44 petroleum ether/AcOEt: 7/3 v/v; isolated yield 66 % (25 mg). ^{1}H NMR (300 MHz, $CDCl_3$): δ = 7.79 (d, 2H, arom CH, $^{3}J_{HH}$ = 8.4 Hz), 7.44 (d, 2H, arom CH, $^{3}J_{HH}$ = 9.0 Hz), 7.40 (d, 1H, arom CH, $^{3}J_{HH}$ = 8.0 Hz), 7.33-7.30 (m, 3H, arom CH), 7.20 (d, 1H, arom CH, $^{3}J_{HH}$ = 8.1 Hz), 6.95 (d, 2H, arom CH, $^{3}J_{HH}$ = 8.7 Hz), 4.66 (d, 2H, NCH_2, $^{4}J_{HH}$ = 3.6 Hz), 4.64 (d, 2H, NCH_2, $^{4}J_{HH}$ = 3.6 Hz), 3.84 (s, 3H, OCH_3), 2.40 (s, 3H, CH_3 of tosyl); $^{13}C\{^{1}H\}$ NMR (126 MHz, $CDCl_3$): δ = 159.50, 143.84, 141.02, 136.96, 134.63, 133.87, 133.210 (7s, arom Cquat), 129.98, 128.28, 127.78, 126.63, 123.02, 121.01, 114.43 (7s, arom CH), 55.51 (s, OCH_3), 53.89 (s, NCH_2), 53.69 (s, NCH_2), 21.66 (s, CH_3 of tosyl) ppm. Consistent with the literature data F. Xu, X.-J. Si, X.-N. Wang, H.-D. Kou, D.-M. Chen, C.-S. Liu, M. Du, *RSC Adv.* **2018**, *8*, 4895-4899.

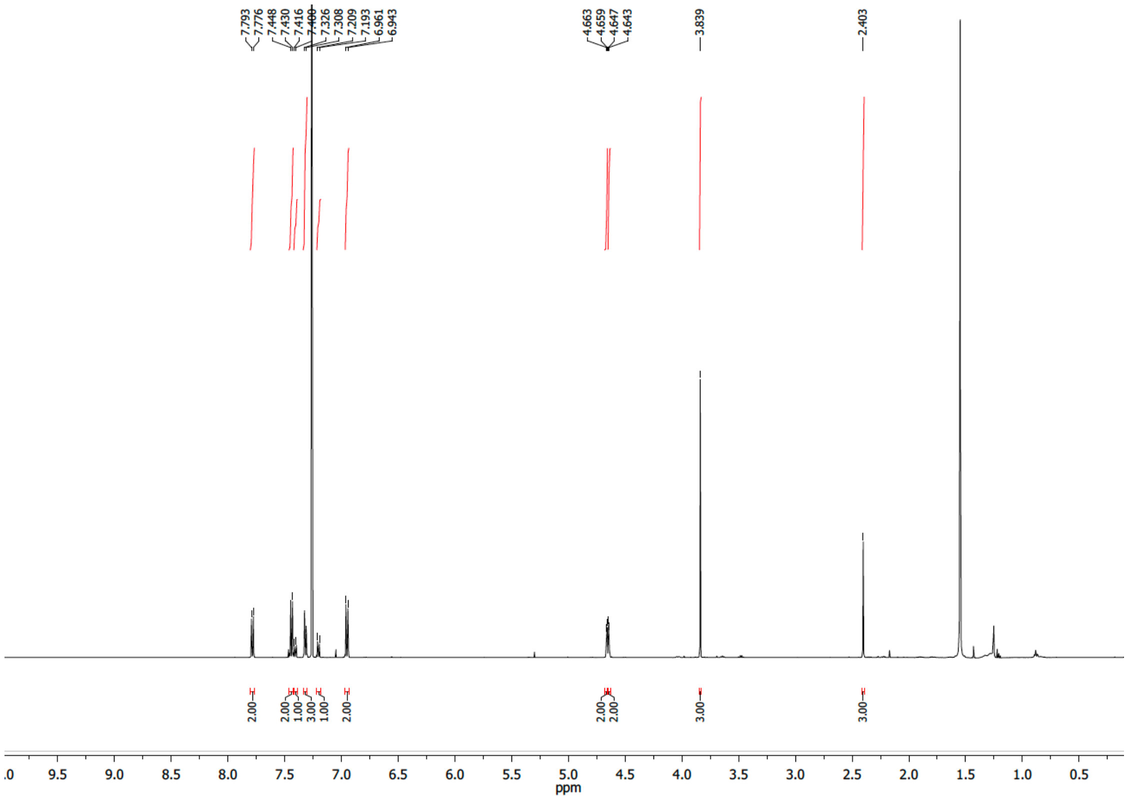

Figure S10. ^{1}H NMR spectrum ($CDCl_3$)

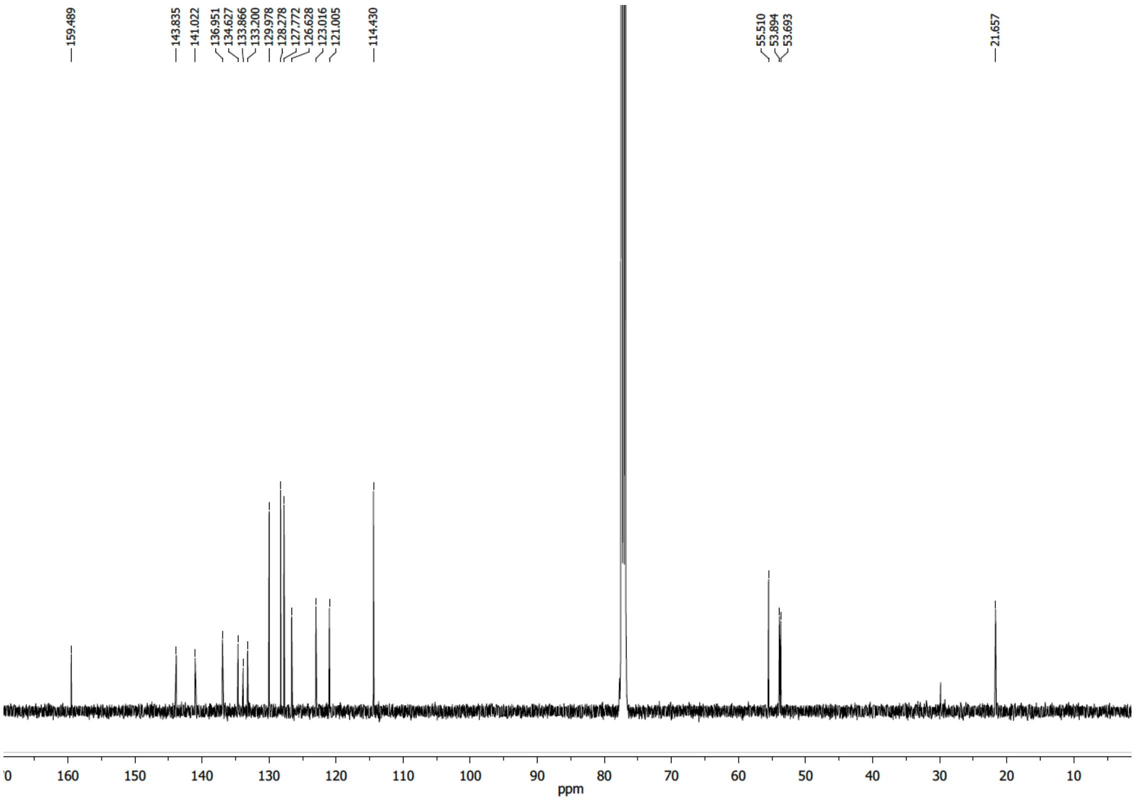

Figure S11. $^{13}C\{^{1}H\}$ NMR spectrum ($CDCl_3$)

5-(4-(*tert*-Butyl)phenyl)-2-tosylisoindoline (6d)

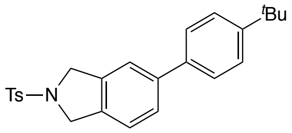

*R*f: 0.83 petroleum ether/AcOEt: 7/3 v/v; isolated yield 52 % (21 mg). ^{1}H NMR (300 MHz, $CDCl_3$): δ = 7.79 (d, 2H, arom CH, $^{3}J_{HH}$ = 8.4 Hz), 7.46-7.43 (m, 5H, arom CH), 7.36-7.31 (m, 3H, arom CH), 7.22 (d, 1H, arom CH, $^{3}J_{HH}$ = 8.1 Hz), 4.67 (d, 2H, NCH_2, $^{4}J_{HH}$ = 3.6 Hz), 4.65 (d, 2H, CH_2, $^{4}J_{HH}$ = 3.6 Hz), 2.41 (s, 3H, CH_3 of tosyl), 1.35 (s, 9H, $C(CH_3)_3$); $^{13}C\{^{1}H\}$ NMR (126 MHz, $CDCl_3$): δ = 150.80, 143.84, 141.26, 137.77, 136.94, 135.00, 133.89 (7s, arom Cquat), 129.99, 127.79, 126.91 126.89, 125.96, 123.02, 121.29 (7s, arom CH), 53.90 (s, NCH_2), 53.71 (s, NCH_2), 34.71 (s, *C*$(CH_3)_3$), 31.48 (s, C(*C*$H_3)_3$), 21.66 (s, CH_3 of tosyl) ppm.

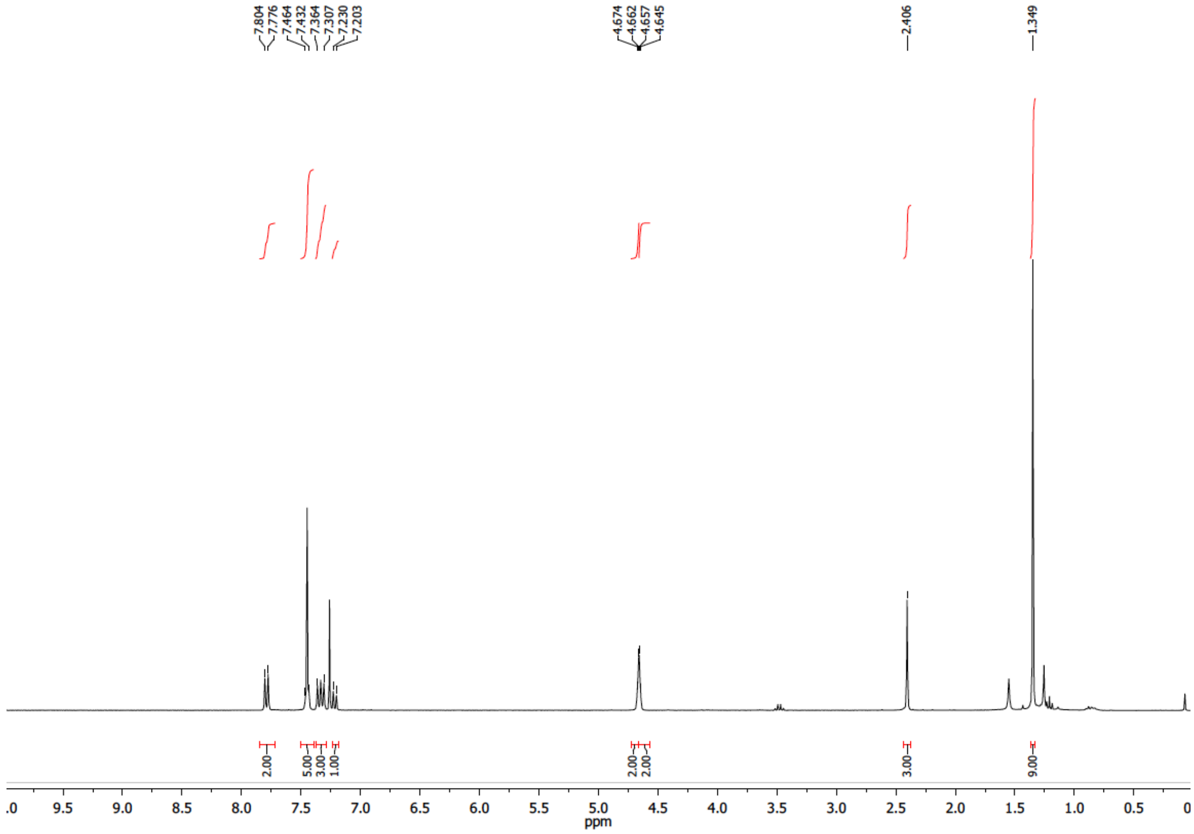

Figure S12. ^{1}H NMR spectrum ($CDCl_3$)

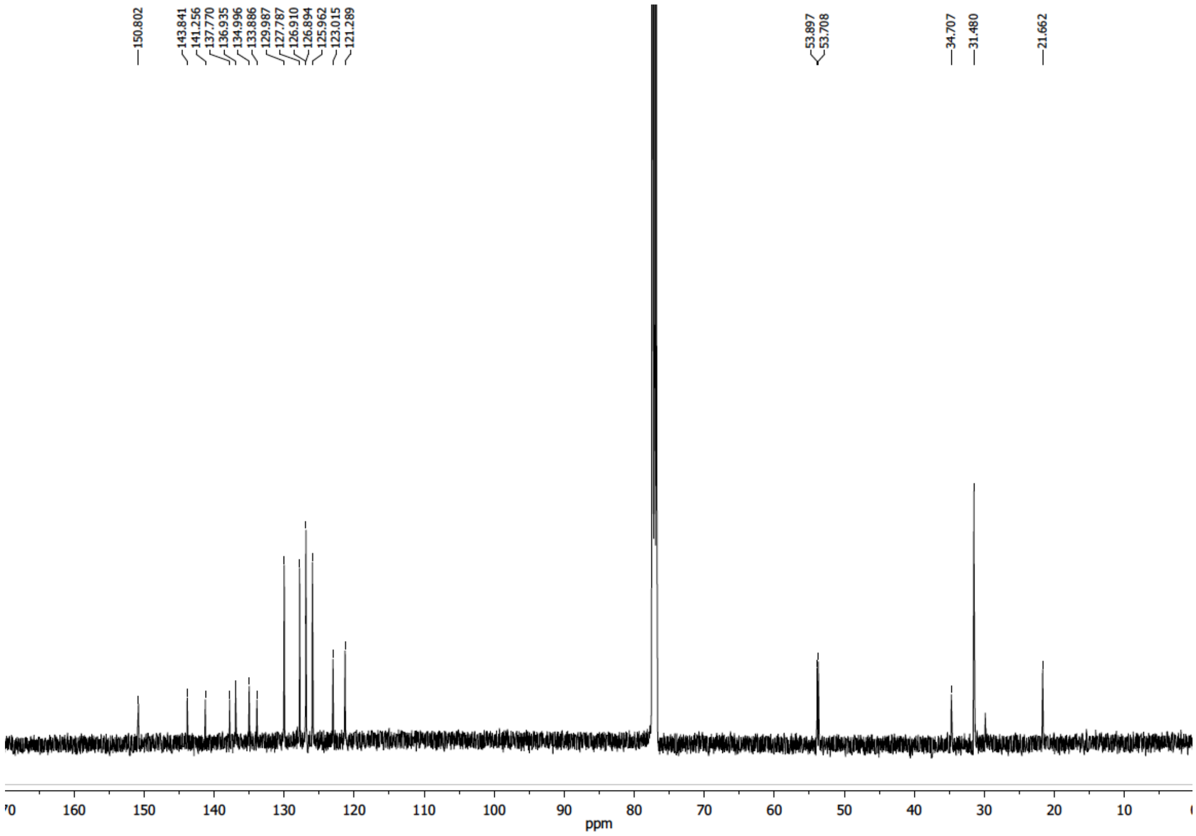

Figure S13. $^{13}C\{^{1}H\}$ NMR spectrum ($CDCl_3$)

5-(4-Chlorophenyl)-2-tosylisoindoline (6e)

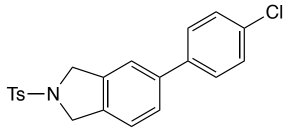

Rf: 0.71 petroleum ether/AcOEt: 7/3 v/v; isolated yield 55 % (21 mg). ^{1}H NMR (500 MHz, $CDCl_3$): δ = 7.79 (d, 2H, arom CH, $^{3}J_{HH}$ = 8.5 Hz), 7.45-7.37 (m, 5H, arom CH), 7.33-7.31 (m, 3H, arom CH), 7.23 (d, 1H, arom CH, $^{3}J_{HH}$ = 8.0 Hz), 4.67 (d, 2H, NCH_2, $^{4}J_{HH}$ = 2.5 Hz), 4.65 (d, 2H, NCH_2, $^{4}J_{HH}$ = 2.5 Hz), 2.41 (s, 3H, CH_3 of tosyl); $^{13}C\{^{1}H\}$ NMR (126 MHz, $CDCl_3$): δ = 143.90, 140.16, 139.13, 137.17, 135.68, 133.84, 133.82 (7s, arom Cquat), 130.00, 129.15, 128.48, 127.76, 126.88, 123.21, 121.32 (7s, arom. CH), 53.82 (s, NCH_2), 53.66 (s, NCH_2), 21.66 (s, CH_3 oftosyl) ppm. Consistent with the literature data Z. Li, Y. Mao, Y. Sun, B. Ma, Y. Wang, G. Zhou, Y. Zhang, F. Zeng, Z. Wang, B. Li, *Results Chem*. **2024**, *7*, 101523.

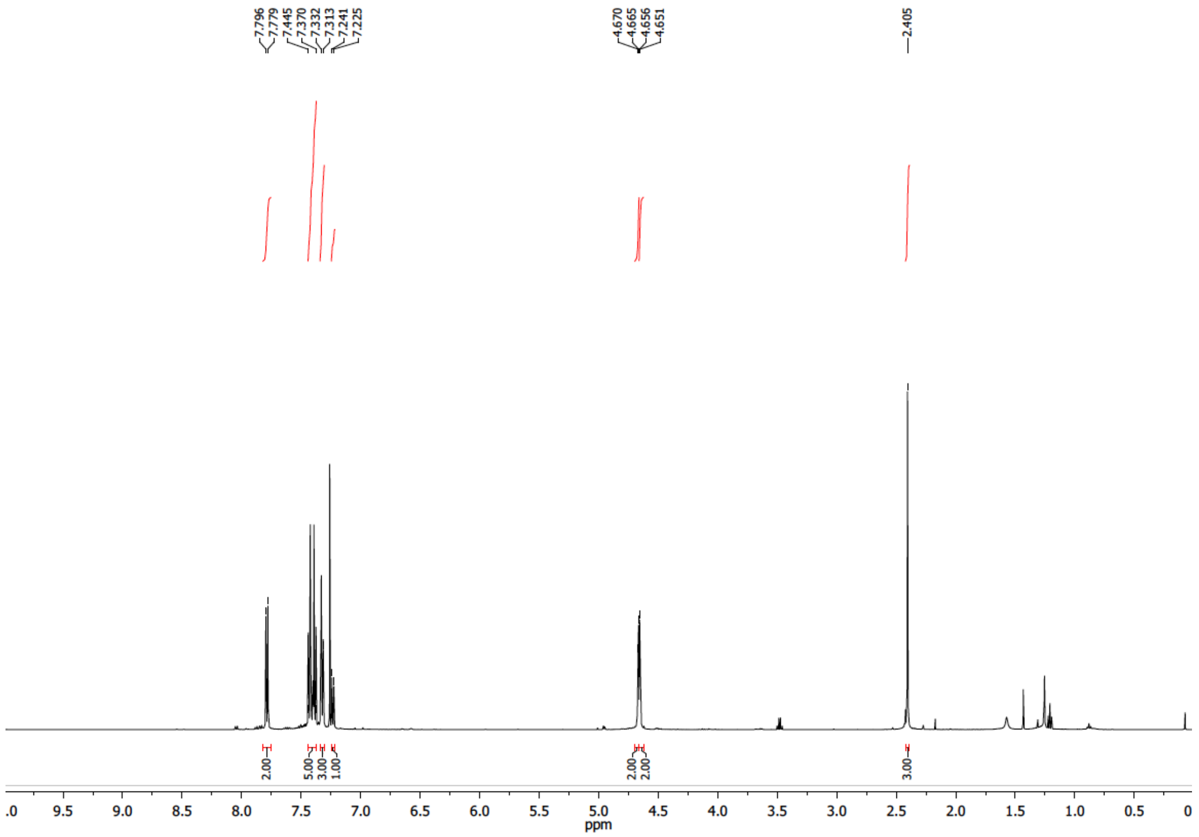

Figure S14. ^{1}H NMR spectrum ($CDCl_3$)

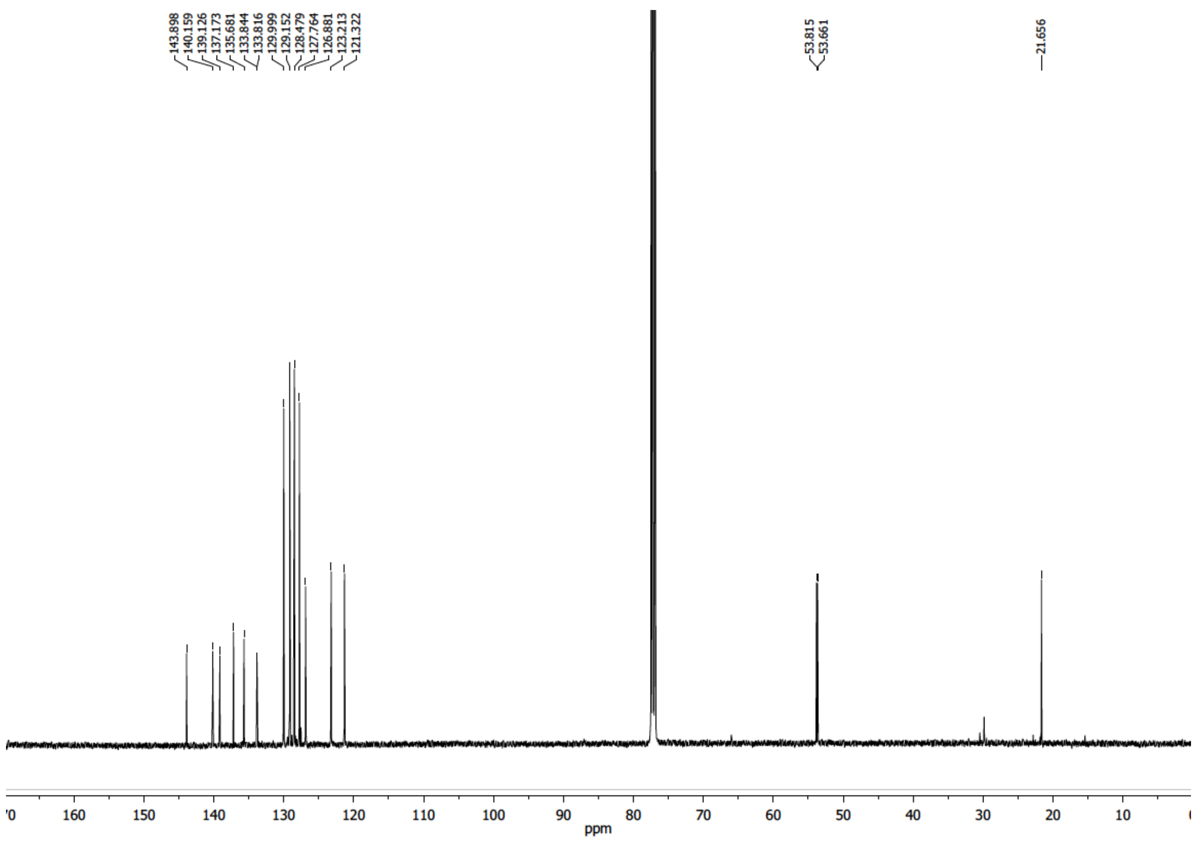

Figure S15. $^{13}C\{^{1}H\}$ NMR spectrum ($CDCl_3$)

5-(4-Fluorophenyl)-2-tosylisoindoline (6f)

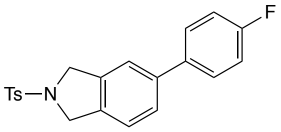

Rf: 0.69 petroleum ether/AcOEt: 7/3 v/v; isolated yield 58 % (21 mg). ^{1}H NMR (500 MHz, $CDCl_3$): δ = 7.79 (d, 2H, arom CH, $^{3}J_{HH}$ = 8.0 Hz), 7.47-7.44 (m, 2H, arom CH), 7.40 (dd, 1H, arom CH, $^{3}J_{HH}$ = 8.0 Hz, $^{4}J_{HH}$ = 2.0 Hz), 7.33-7.31 (m, 3H, arom CH), 7.23 (d, 1H, arom CH, $^{3}J_{HH}$ = 7.5 Hz), 7.12-7.09 (m, 2H, arom CH), 4.67 (d, 2H, NCH_2, $^{4}J_{HH}$ = 2.5 Hz), 4.65 (d, 2H, NCH_2, $^{4}J_{HH}$ = 2.5 Hz), 2.41 (s, 3H, CH_3 of tosyl); ^{13}C{^{1}H} NMR (126 MHz, $CDCl_3$): δ = 162.72 (d, CF, $^{1}J_{CF}$ = 247.6 Hz), 143.89, 140.43, 137.09, 135.32, 133.84 (5s, arom Cquat), 136.82 (d, arom Cquat of C_6H_4F, $^{4}J_{CF}$ = 3.3 Hz), 130.00, 127.77, 126.92, 123.14, 121.36 (5s, arom CH), 128.82 (d, arom CH of C_6H_4F, $^{3}J_{CF}$ = 8.1 Hz), 115.89 (d, arom CH of C_6H_4F, $^{2}J_{CF}$ = 21.4 Hz), 53.83 (s, NCH_2), 53.66 (s, NCH_2), 21.66 (s, CH_3 of tosyl); ^{19}F{^{1}H} NMR (282 MHz, $CDCl_3$): δ = -115.21 (s, CF) ppm. Consistent with the literature data Z. Li, Y. Mao, Y. Sun, B. Ma, Y. Wang, G. Zhou, Y. Zhang, F. Zeng, Z. Wang, B. Li, *Results Chem*. **2024**, *7*, 101523.

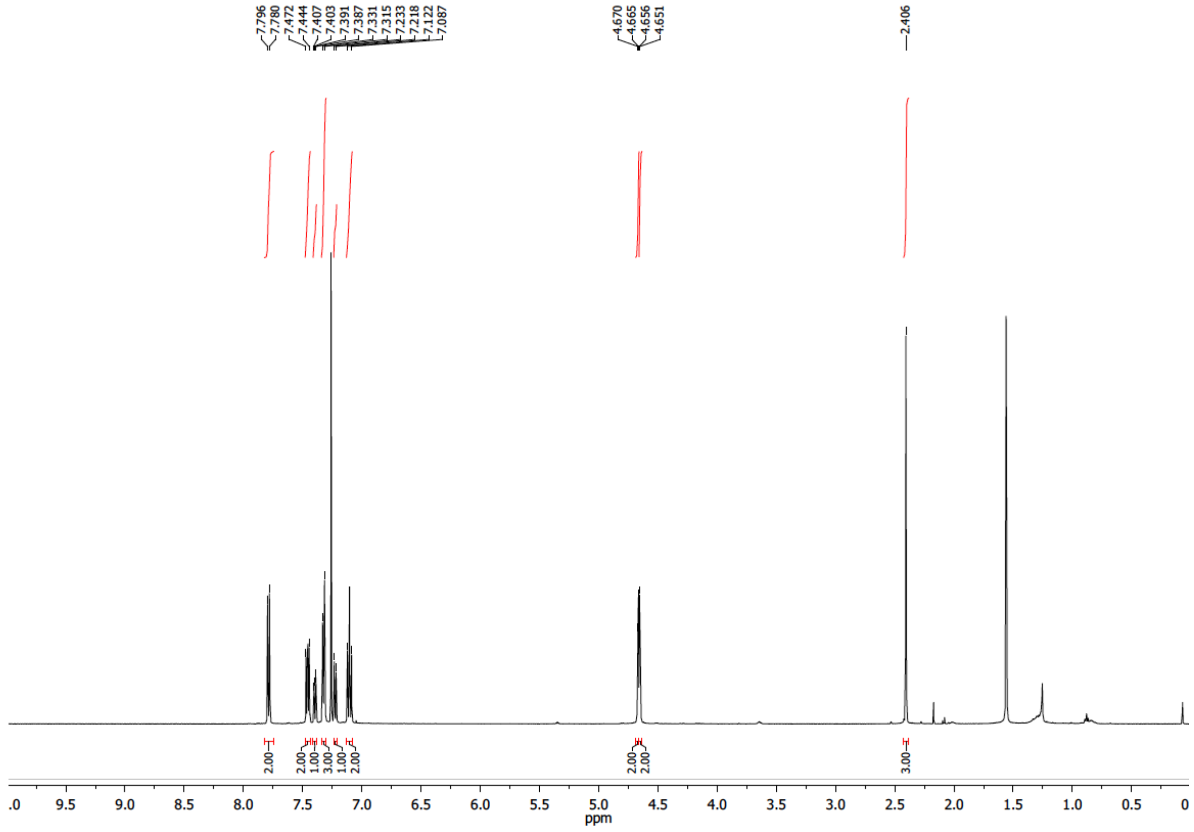

Figure S16. ^{1}H NMR spectrum ($CDCl_3$)

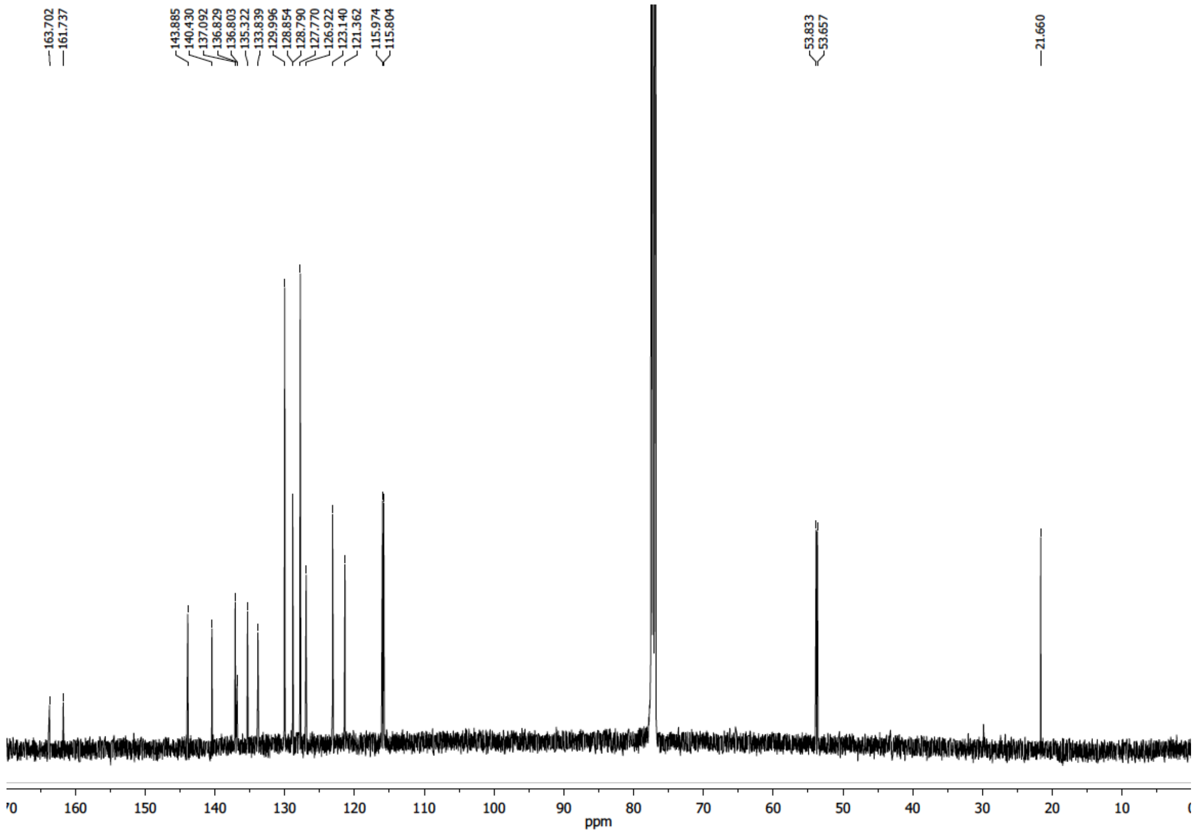

Figure S17. $^{13}C\{^1H\}$ NMR spectrum ($CDCl_3$)

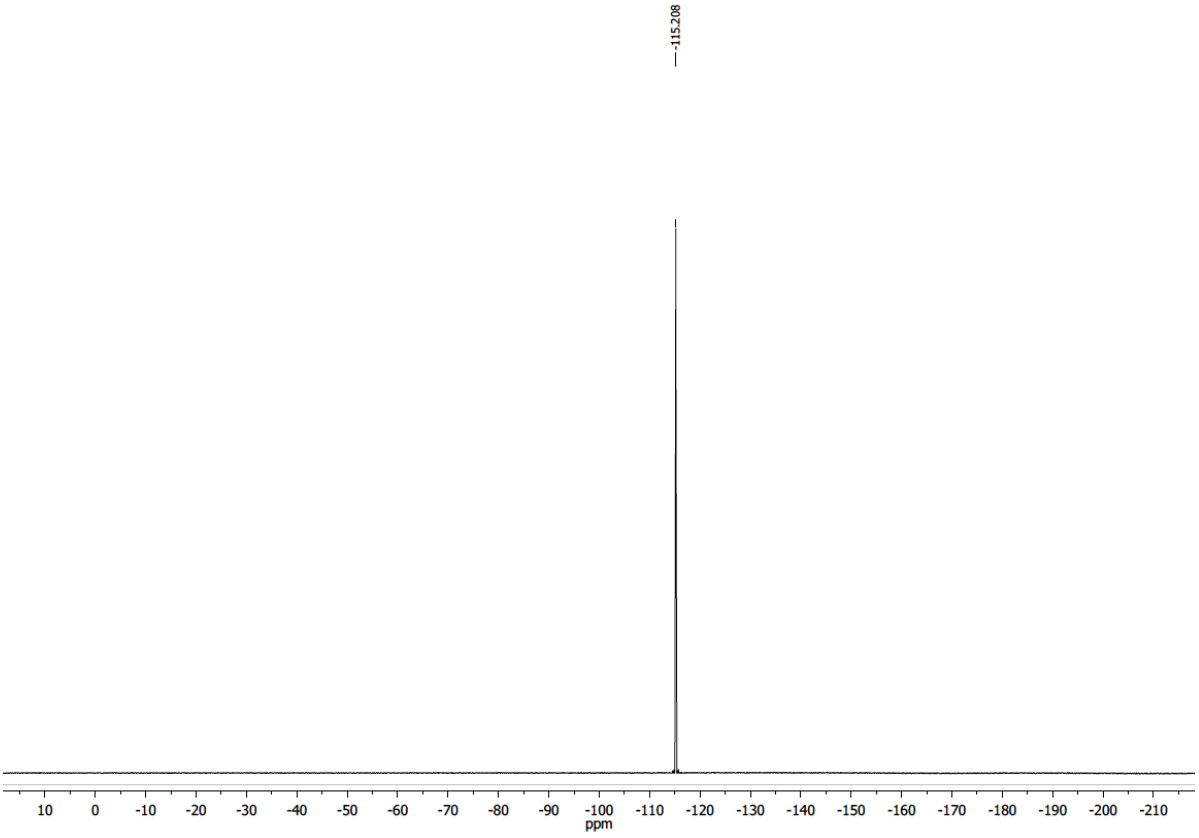

Figure S18. $^{19}F\{^1H\}$ NMR spectrum ($CDCl_3$)

5-(*o*-Tolyl)-2-tosylisoindoline (6g)

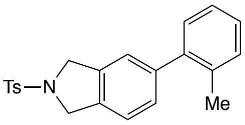

Rf: 0.69 petroleum ether/AcOEt: 7/3 v/v; isolated yield 52 % (19 mg). ^{1}H NMR (500 MHz, $CDCl_3$): δ = 7.80 (d, 2H, arom CH, $^{3}J_{HH}$ = 8.5 Hz), 7.33 (d, 2H, arom CH, $^{3}J_{HH}$ = 8.0 Hz), 7.26-7.17 (m, 5H, arom CH), 7.14-7.11 (m, 2H, arom CH), 4.67 (d, 2H, NCH_2, $^{4}J_{HH}$ = 2.5 Hz), 4.66 (d, 2H, NCH_2, $^{4}J_{HH}$ = 2.5 Hz), 2.42 (s, 3H, CH_3 of tosyl), 2.21 (s, 3H, CH_3); $^{13}C\{^{1}H\}$ NMR (126 MHz, $CDCl_3$): δ = 143.82, 141.94, 141.31, 136.31, 135.36, 134.81, 133.94 (7s, arom Cquat), 130.52, 129.98, 129.83, 128.97, 127.82, 127.66, 125.98, 123.44, 122.40 (9s, arom CH), 53.84 (s, NCH_2), 53.73 (s, NCH_2), 21.67 (s, CH_3 of tosyl), 20.55 (s, CH_3) ppm. Consistent with the literature data Z. Li, Y. Mao, Y. Sun, B. Ma, Y. Wang, G. Zhou, Y. Zhang, F. Zeng, Z. Wang, B. Li, *Results Chem*. **2024**, *7*, 101523.

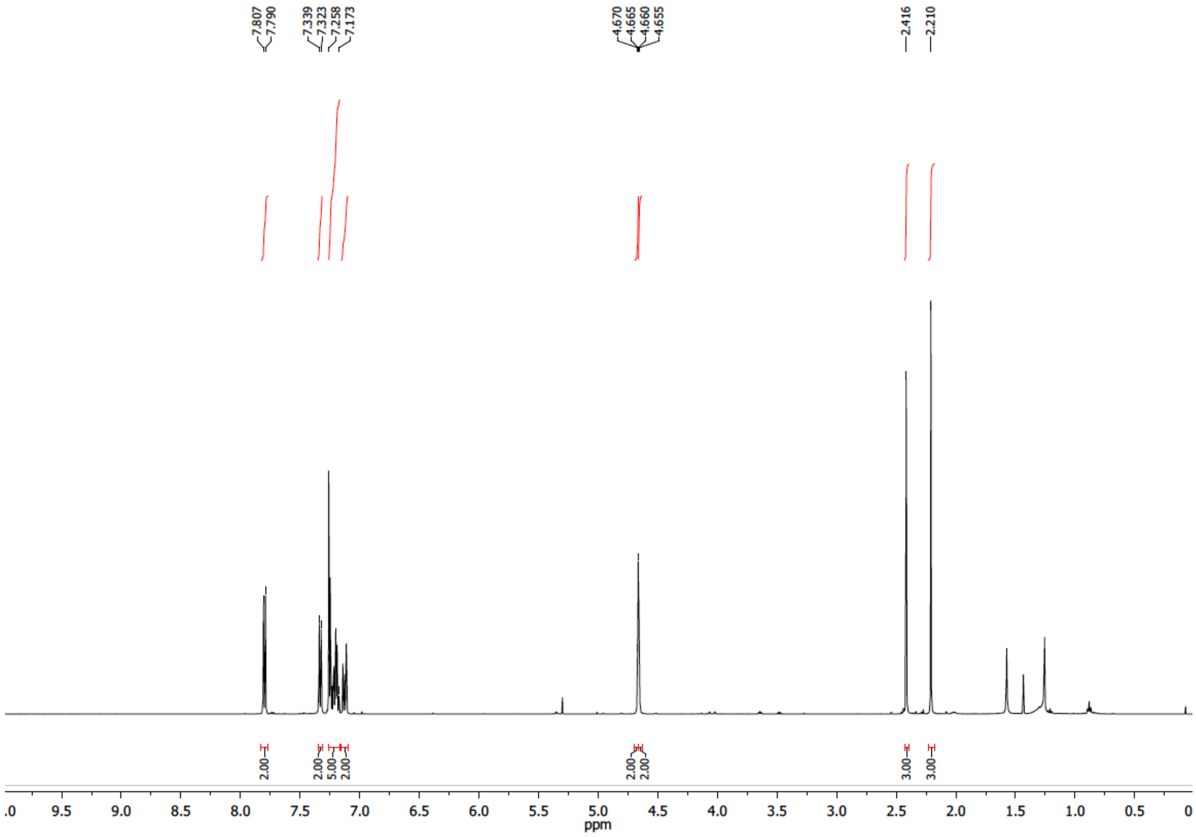

Figure S19. ^{1}H NMR spectrum ($CDCl_3$)

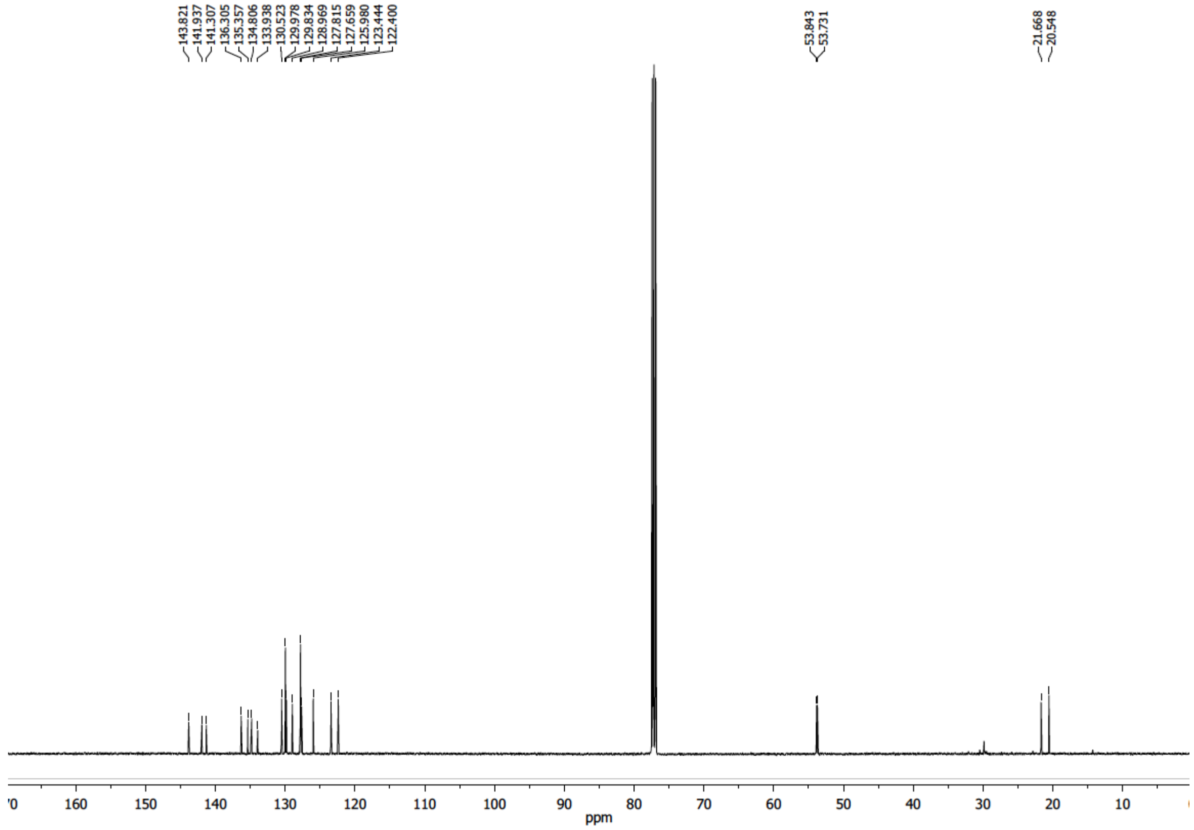

Figure S20. $^{13}C\{^{1}H\}$ NMR spectrum ($CDCl_3$)

5-(2-Fluorophenyl)-2-tosylisoindoline (6h)

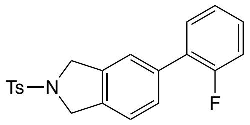

*R*f: 0.67 petroleum ether/AcOEt: 7/3 v/v; isolated yield 49 % (18 mg). ^{1}H NMR (500 MHz, $CDCl_3$): δ = 7.79 (d, 2H, arom CH, $^3J_{HH}$ = 8.0 Hz), 7.40 (d, 1H, arom CH, $^3J_{HH}$ = 8.0 Hz), 7.37-7.29 (m, 5H, arom CH), 7.24 (d, 1H, arom CH, $^3J_{HH}$ = 8.0 Hz), 7.19 (ddd, 1H, arom CH, $^3J_{HH}$ = 7.5 Hz, $^3J_{HH}$ = 7.5 Hz, $^4J_{HH}$ = 1.5 Hz), 7.13 (ddd, 1H, arom. CH, $^3J_{HH}$ = 8.5 Hz, $^3J_{HH}$ = 8.0 Hz, $^4J_{HH}$ = 1.5 Hz), 4.67 (d, 2H, NCH_2, $^4J_{HH}$ = 3.0 Hz), 4.66 (d, 2H, NCH_2, $^4J_{HH}$ = 3.0 Hz), 2.41 (s, 3H, CH_3 of tosyl); ^{13}C{^{1}H} NMR (126 MHz, $CDCl_3$): δ = 159.78 (d, CF, $^1J_{CF}$ = 248.1 Hz), 143.89, 136.66, 135.75, 135.74, 133.81 (5s, arom Cquat), 130.79 (d, arom CH, $^4J_{CF}$ = 3.3 Hz), 130.00 (s, arom CH), 129.43 (d, arom CH of C_6H_4F, $^3J_{CF}$ = 8.2 Hz), 128.82 (d, arom CH, $^4J_{CF}$ = 2.6 Hz), 128.54 (d, arom Cquat of C_6H_4F, $^2J_{CF}$ = 13.5 Hz), 127.78 (s, arom CH), 124.59 (d, arom CH of C_6H_4F, $^3J_{CF}$ = 3.7 Hz), 123.39 (d, arom CH, $^4J_{CF}$ = 3.0 Hz), 122.81 (s, arom CH), 116.28 (d, arom CH of C_6H_4F, $^2J_{CF}$ = 22.7 Hz), 53.86 (s, NCH_2), 53.76 (s, NCH_2), 21.66 (s, CH_3 of tosyl); ^{19}F{^{1}H} NMR (282 MHz, $CDCl_3$): δ = -118.17 (s, CF) ppm. MS (ESI-MS): m/z = 309.0950 $[M + Na]^+$ (calcd. for $C_{21}H_{18}NSO_2FNa$: 390.0934).

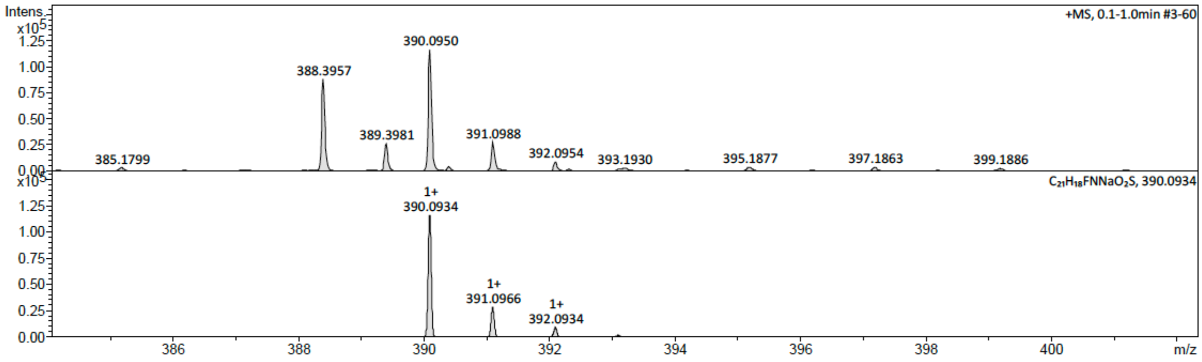

Figure S21. Mass spectrum (ESI-MS)
exp. spectrum (top); calc. spectrum (bottom) for $C_{21}H_{18}NSO_2FNa$ $[M + Na]^+$

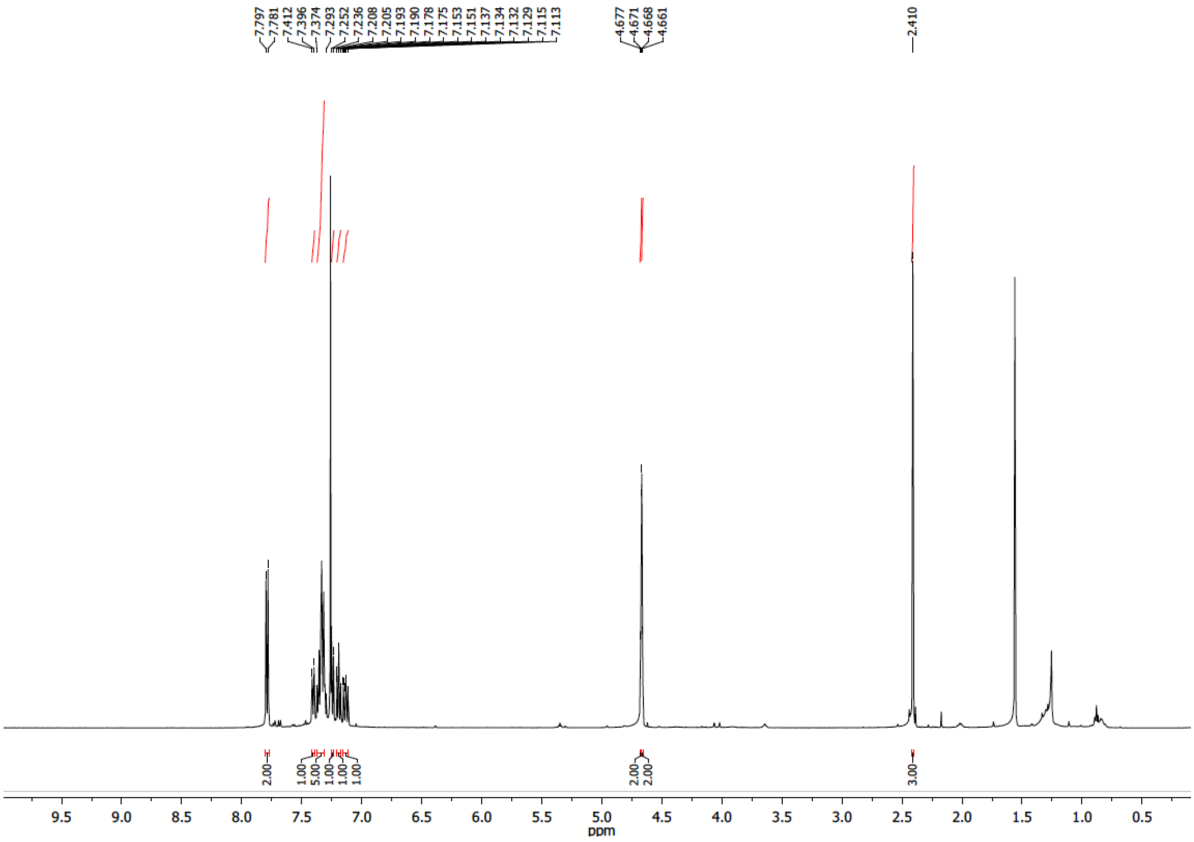

Figure S22. ^{1}H NMR spectrum ($CDCl_3$)

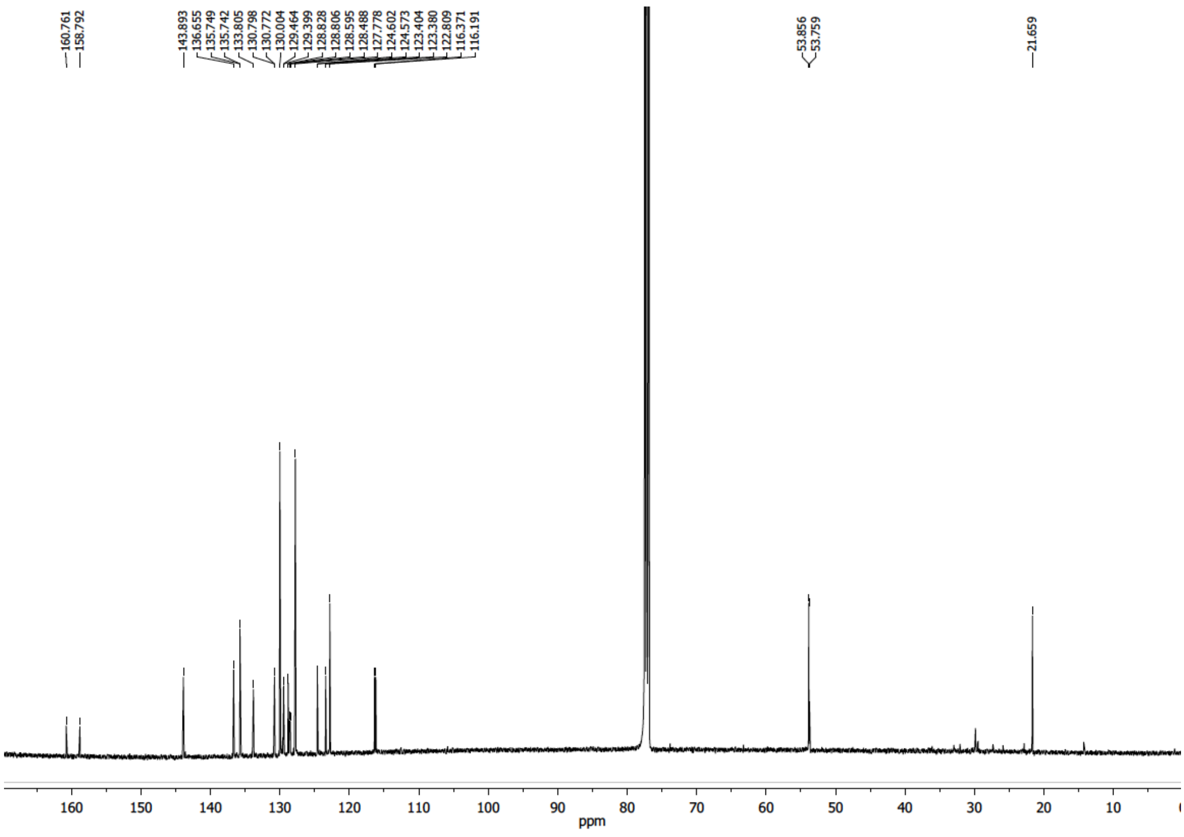

Figure S23. $^{13}C\{^{1}H\}$ NMR spectrum ($CDCl_3$)

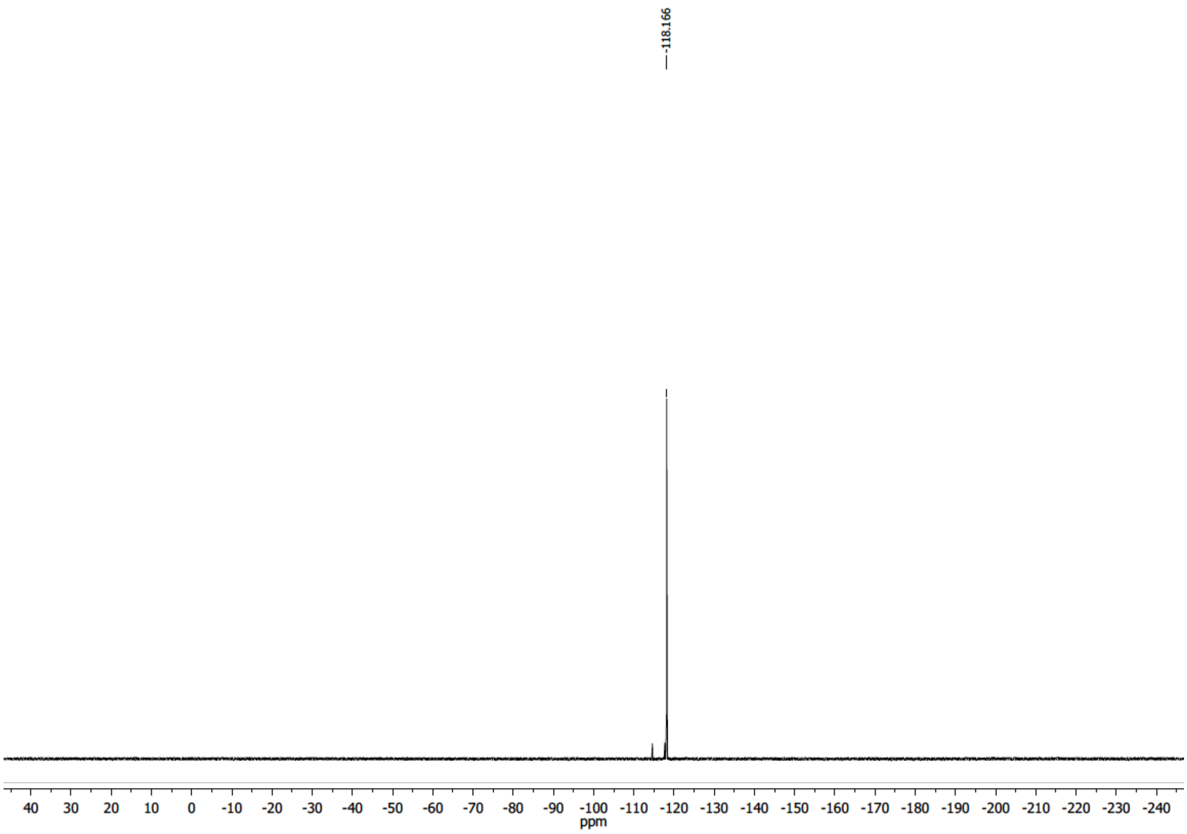

Figure S24. $^{19}F\{^1H\}$ NMR spectrum ($CDCl_3$)

5-(Naphthalen-2-yl)-2-tosylisoindoline (6i)

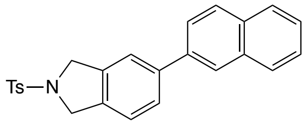

*R*f: 0.76 petroleum ether/AcOEt: 7/3 v/v; isolated yield 58 % (23 mg). ^{1}H NMR (500 MHz, $CDCl_3$): δ = 7.96 (s, 1H, arom CH), 7.91-7.85 (m, 3H, arom CH), 7.80 (d, 2H, arom CH, $^{3}J_{HH}$ = 8.0 Hz), 7.65 (dd, 1H, arom CH, $^{3}J_{HH}$ = 8.5 Hz, $^{4}J_{HH}$ = 2.0 Hz), 7.59 (dd, 1H, arom CH, $^{3}J_{HH}$ = 8.0 Hz, $^{4}J_{HH}$ = 1.5 Hz), 7.51-7.47 (m, 3H, arom CH), 7.33 (d, 2H, arom CH, $^{3}J_{HH}$ = 8.0 Hz), 7.28 (d, 1H, arom CH, $^{3}J_{HH}$ = 8.0 Hz), 4.71 (d, 2H, NCH_2, $^{4}J_{HH}$ = 1.5 Hz), 4.69 (d, 2H, NCH_2, $^{4}J_{HH}$ =1.5 Hz), 2.41 (s, 3H, CH_3 of tosyl); $^{13}C\{^{1}H\}$ NMR (126 MHz, $CDCl_3$): δ = 143.88, 141.33, 137.99, 137.13, 135.40, 133.87, 133.74, 132.83 (8s, arom Cquat), 130.01, 128.72, 128.30, 127.80, 127.79, 127.33, 126.61, 126.29, 126.00, 125.52, 123.18, 121.73 (12s, arom CH), 53.91 (s, NCH_2), 53.74 (s, NCH_2), 21.67 (s, CH_3 of tosyl) ppm. MS (ESI-MS): m/z = 422.1193 $[M + Na]^+$ (calcd. for $C_{25}H_{21}NSO_2Na$: 422.1185).

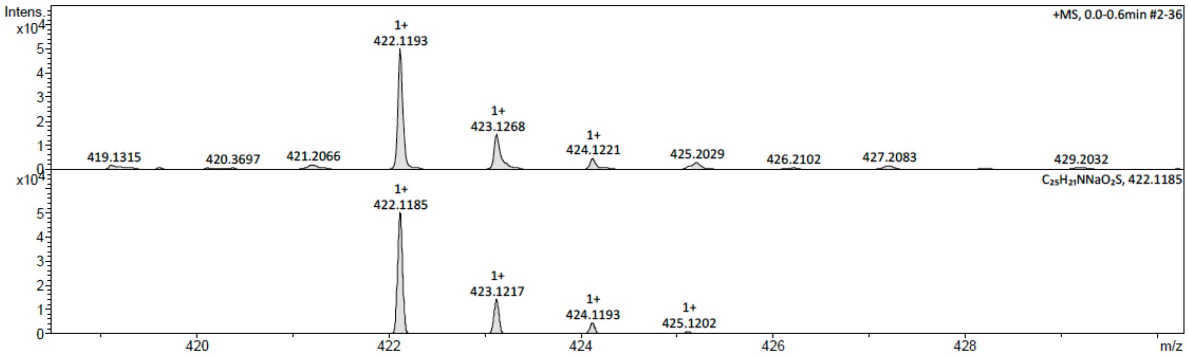

Figure S25. Mass spectrum (ESI-MS)
exp. spectrum (top); calc. spectrum (bottom) for $C_{25}H_{21}NSO_2Na[M + Na]^+$

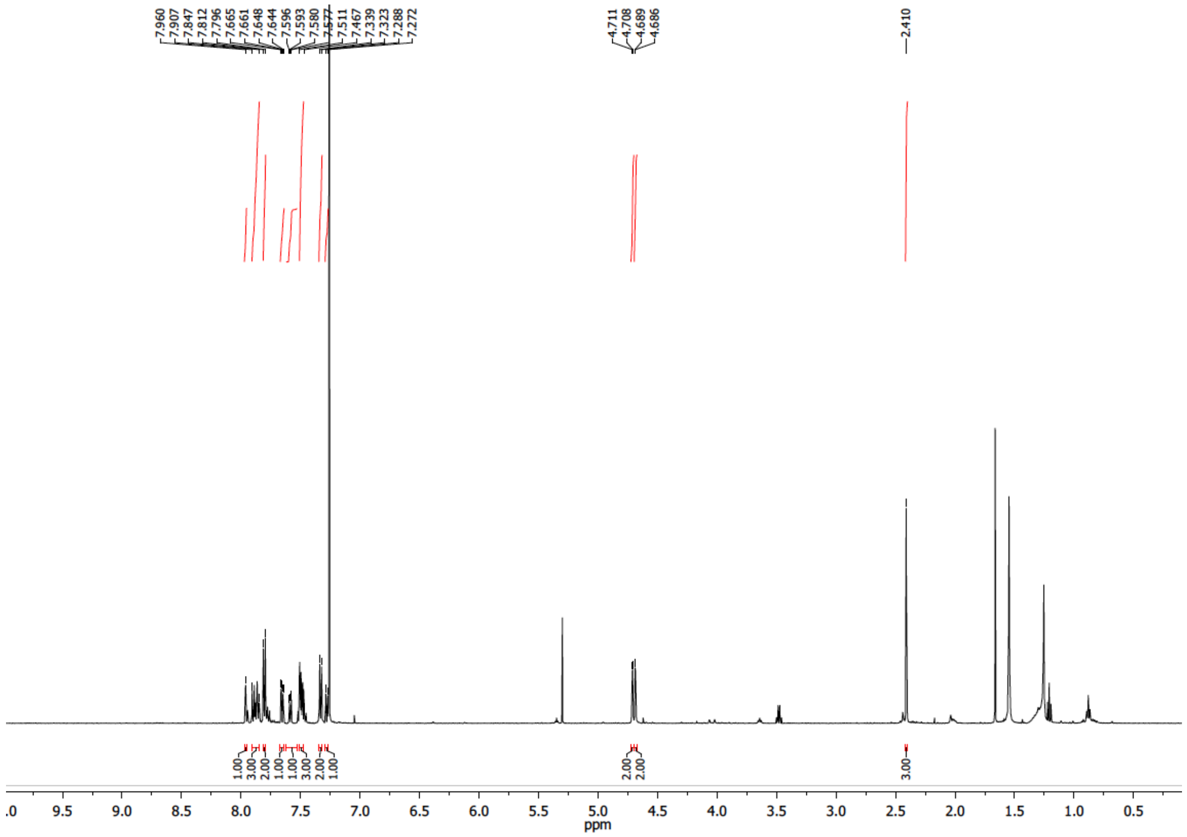

Figure S26. ^{1}H NMR spectrum ($CDCl_3$)

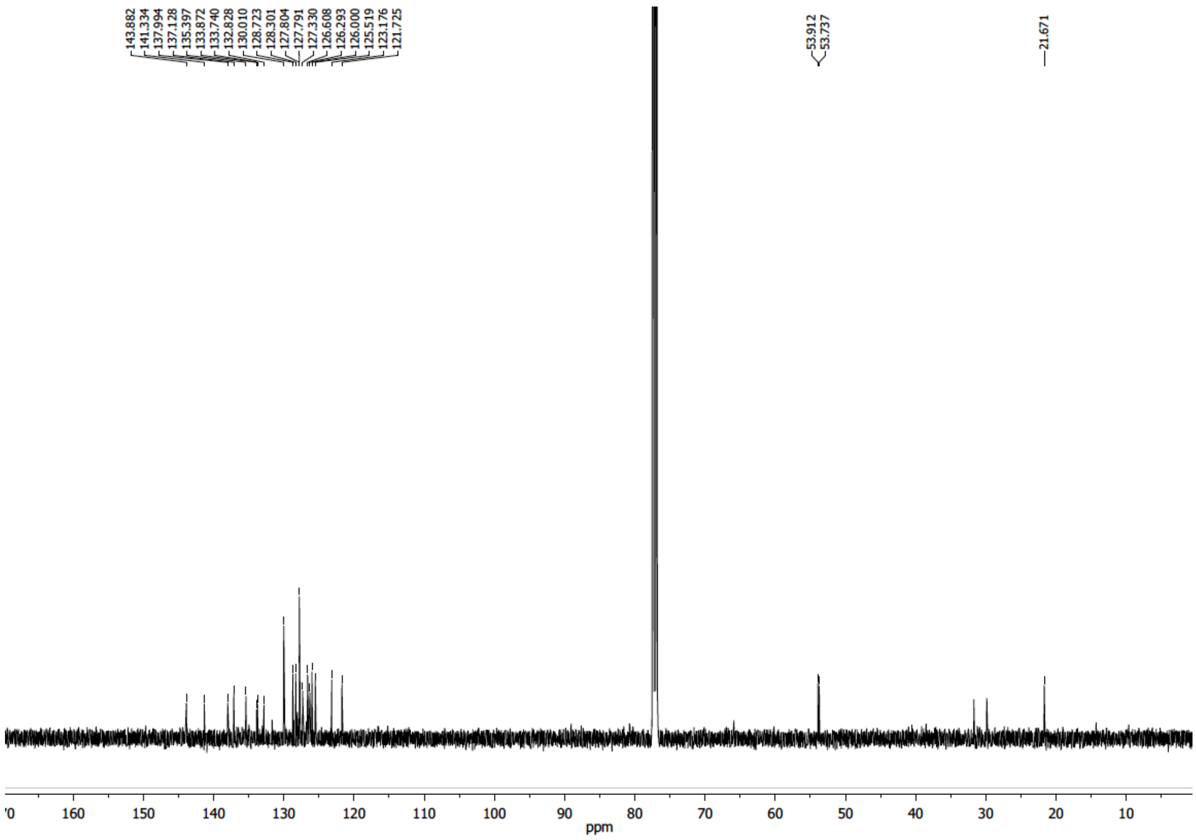

Figure S27. $^{13}C\{^{1}H\}$ NMR spectrum ($CDCl_3$)

5-(6-Methoxynaphthalen-2-yl)-2-tosylisoindoline (6j)

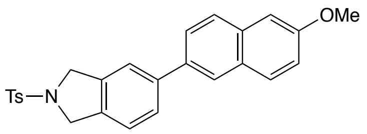

*R*f: 0.57 petroleum ether/AcOEt: 7/3 v/v; isolated yield 60 % (26 mg). ^{1}H NMR (500 MHz, $CDCl_3$): δ = 7.89 (d, 1H, arom CH, $^4J_{HH}$ = 1.5 Hz), 7.80 (d, 2H, arom CH, $^3J_{HH}$ = 8.5 Hz), 7.78 (br s, 1H, arom CH), 7.76 (br s, 1H, arom CH), 7.62 (dd, 1H, arom CH, $^3J_{HH}$ = 8.5 Hz, $^4J_{HH}$ = 2.0 Hz), 7.56 (dd, 1H, arom CH, $^3J_{HH}$ = 8.0 Hz, $^4J_{HH}$ = 1.5 Hz), 7.48 (br s, 1H, arom CH), 7.33 (d, 2H, arom CH, $^3J_{HH}$ = 8.0 Hz), 7.26 (d, 1H, arom CH, $^3J_{HH}$ = 8.5 Hz), 7.17 (dd, 1H, arom CH, $^3J_{HH}$ = 9.0 Hz, $^4J_{HH}$ = 3.0 Hz), 7.15 (d, 1H, arom CH, $^4J_{HH}$ = 2.5 Hz), 4.70 (d, 2H, NCH_2, $^4J_{HH}$ = 1.5 Hz), 4.68 (d, 2H, NCH_2, $^4J_{HH}$ = 1.5 Hz), 3.94 (s, 3H, OCH_3), 2.41 (s, 3H, CH_3 of tosyl); $^{13}C\{^1H\}$ NMR (126 MHz, $CDCl_3$): δ = 158.06, 143.86, 141.41, 137.07, 135.80, 135.06, 134.02, 133.88, 129.23 (9s, arom Cquat), 130.00, 129.81, 127.78, 127.53, 127.11, 125.97, 125.80, 123.12, 121.49, 119.49, 105.70 (11s, arom CH), 55.50 (s, OCH_3), 53.92 (s, NCH_2), 53.73 (s, NCH_2), 21.67 (s, CH_3 of tosyl) ppm. MS (ESI-MS): m/z = 452.1276 $[M + Na]^+$ (calcd. for $C_{26}H_{23}NSO_3Na$: 425.1291).

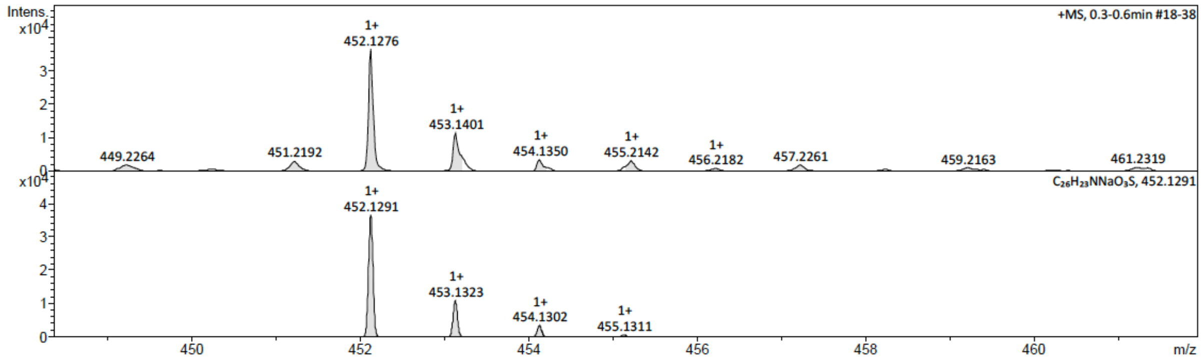

Figure S28. Mass spectrum (ESI-MS)
exp. spectrum (top); calc. spectrum (bottom) for $C_{26}H_{23}NSO_3Na$ $[M + Na]^+$

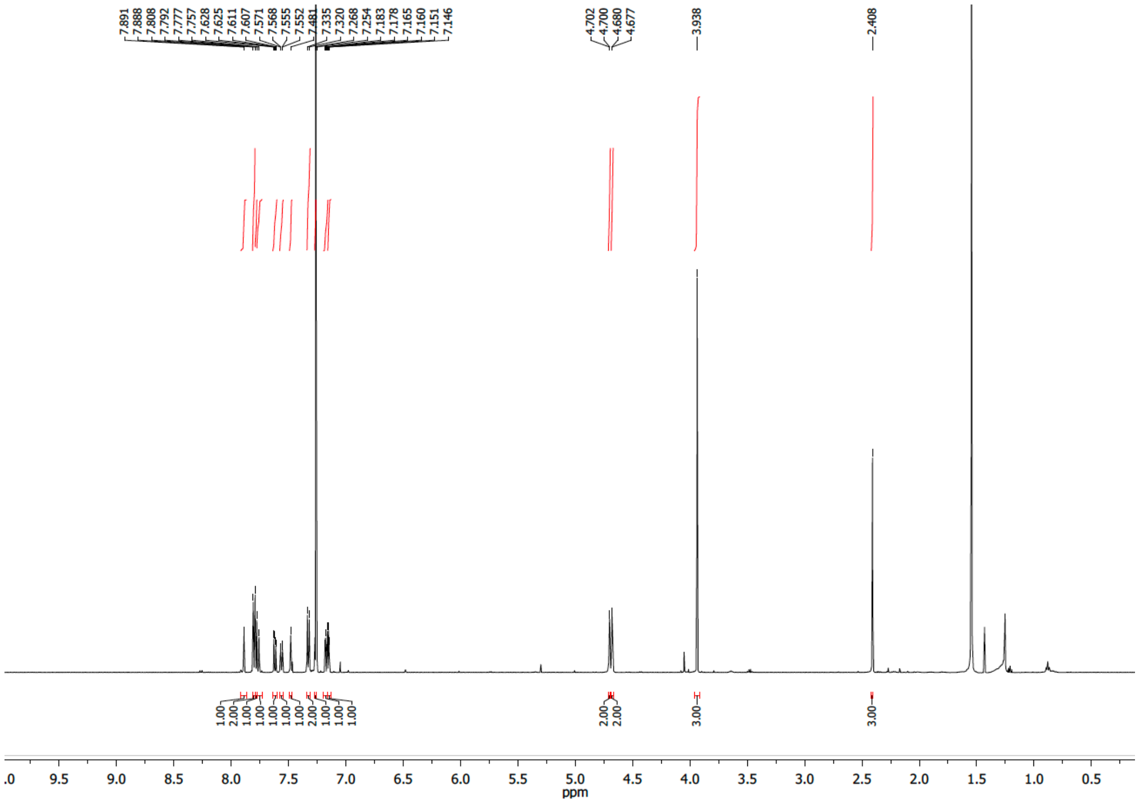

Figure S29. ^{1}H NMR spectrum ($CDCl_3$)

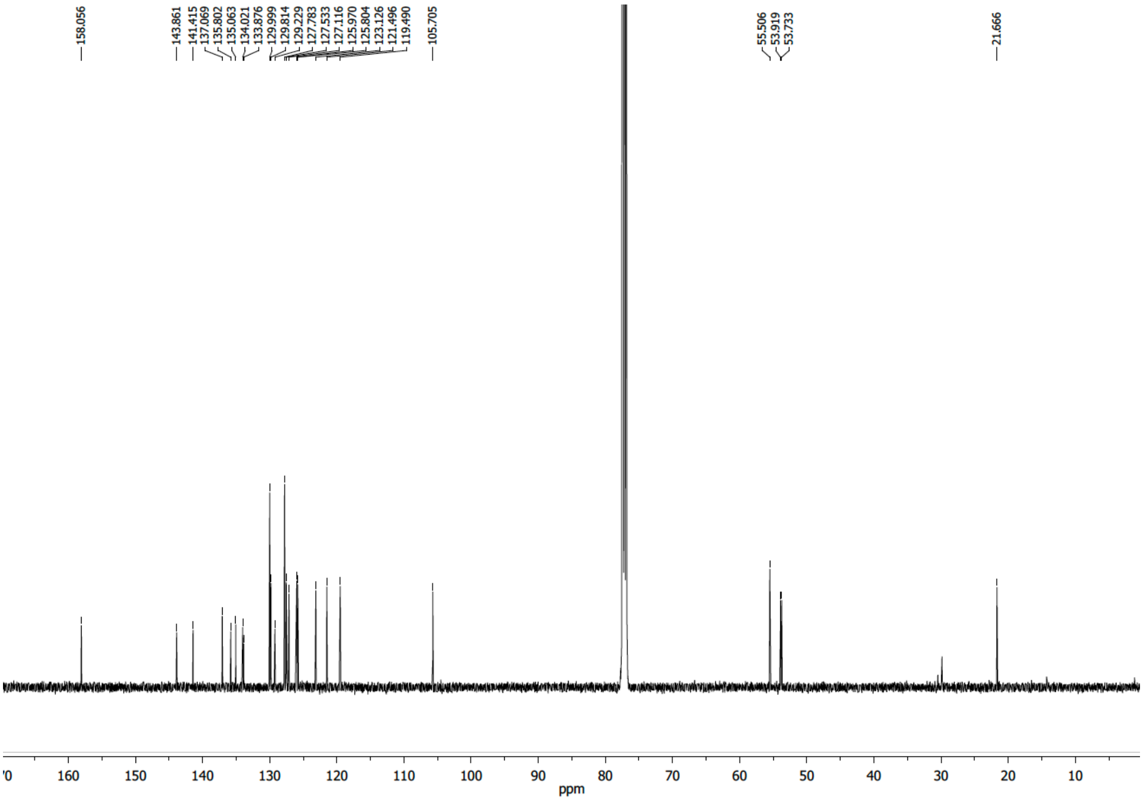

Figure S30. $^{13}C\{^1H\}$ NMR spectrum ($CDCl_3$)

5-(Phenanthren-9-yl)-2-tosylisoindoline (6k)

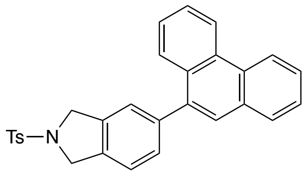

*R*f: 0.70 petroleum ether/AcOEt: 7/3 v/v; isolated yield 47 % (21 mg). ^{1}H NMR (500 MHz, $CDCl_3$): δ = 8.77 (d, 1H, arom CH, $^{3}J_{HH}$ = 8.5 Hz), 8.71 (d, 1H, arom CH, $^{3}J_{HH}$ = 8.0 Hz), 7.86 (dd, 1H, arom CH, $^{3}J_{HH}$ = 8.0 Hz, $^{4}J_{HH}$ = 1.5 Hz), 7.83 (d, 2H, arom CH, $^{3}J_{HH}$ = 8.5 Hz), 7.78 (dd, 1H, arom CH, $^{3}J_{HH}$ = 8.5 Hz, $^{4}J_{HH}$ = 1.0 Hz), 7.69-7.65 (m, 2H, arom CH), 7.63-7.61 (m, 1H, arom CH), 7.60 (s, 1H, arom CH), 7.51 (ddd, 1H, arom CH, $^{3}J_{HH}$ = 8.5 Hz, $^{3}J_{HH}$ = 7.0 Hz, $^{4}J_{HH}$ = 1.5 Hz), 7.41 (dd, 1H, arom CH, $^{3}J_{HH}$ = 8.0 Hz, $^{4}J_{HH}$ = 2.0 Hz), 7.36 (d, 2H, arom CH, $^{3}J_{HH}$ = 8.0 Hz), 7.34 (s, 1H, arom CH), 7.31 (d, 1H, arom CH, $^{3}J_{HH}$ = 8.0 Hz), 4.74 (d, 2H, NCH_2, $^{4}J_{HH}$ = 1.5 Hz), 4.72 (d, 2H, NCH_2, $^{4}J_{HH}$ =1.5 Hz), 2.44 (s, 3H, CH_3 of tosyl); $^{13}C\{^{1}H\}$ NMR (126 MHz, $CDCl_3$): δ = 143.87, 140.78, 138.13, 136.58, 135.46, 133.94, 131.53, 131.08, 130.77, 130.14 (10s, arom Cquat), 130.02, 129.86, 128.79, 127.85, 127.76, 127.10, 126.94, 126.75, 126.72, 126.71, 124.33, 123.14, 122.70, 122.64 (14s, arom CH), 53.88 (s, NCH_2), 53.82 (s, NCH_2), 21.70 (s, CH_3 of tosyl) ppm. MS (ESI-MS): m/z = 472.1325 $[M + Na]^+$ (calcd. for $C_{29}H_{23}NSO_2Na$ 472.1342).

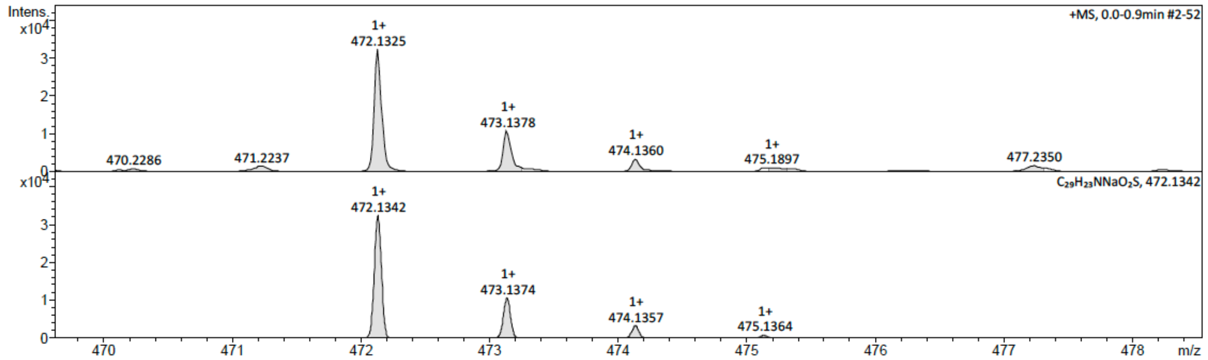

Figure S31. Mass spectrum (ESI-MS)
exp. spectrum (top); calc. spectrum (bottom) for $C_{29}H_{23}NSO_2Na$ $[M + Na]^+$

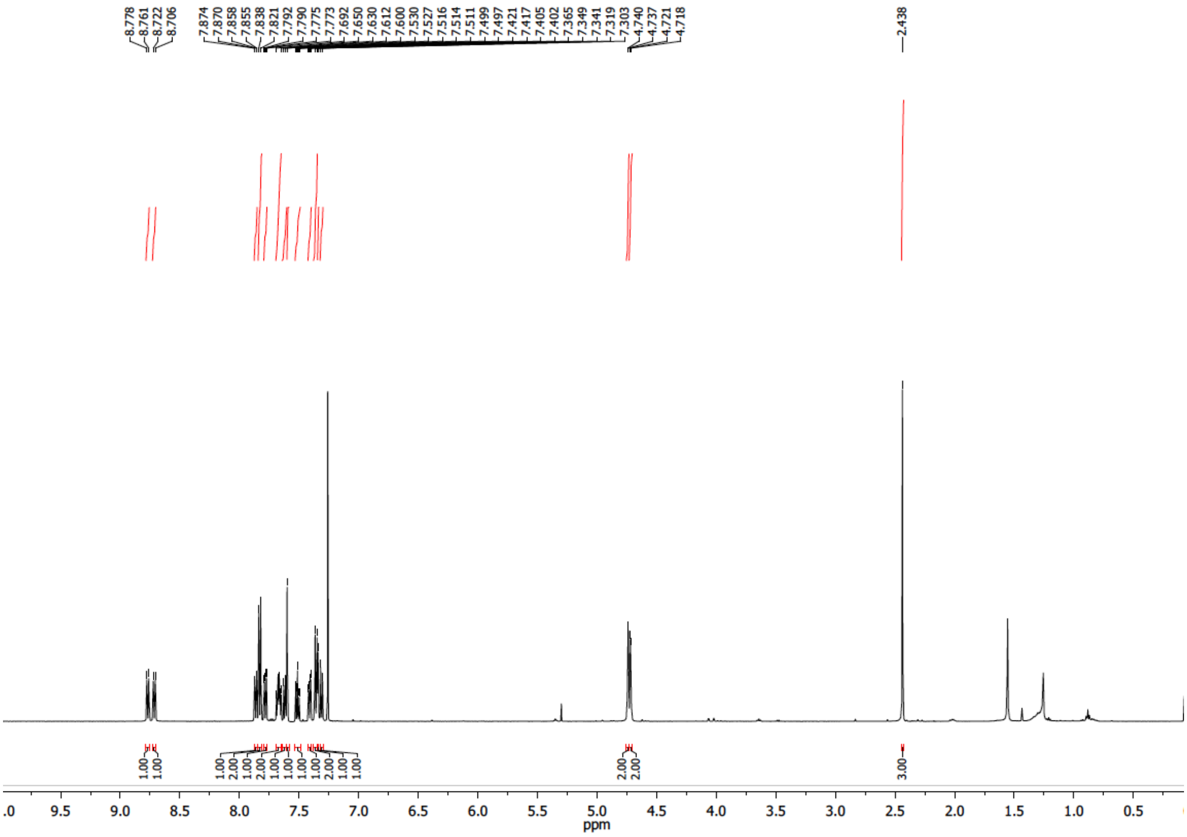

Figure S32. ^{1}H NMR spectrum ($CDCl_3$)

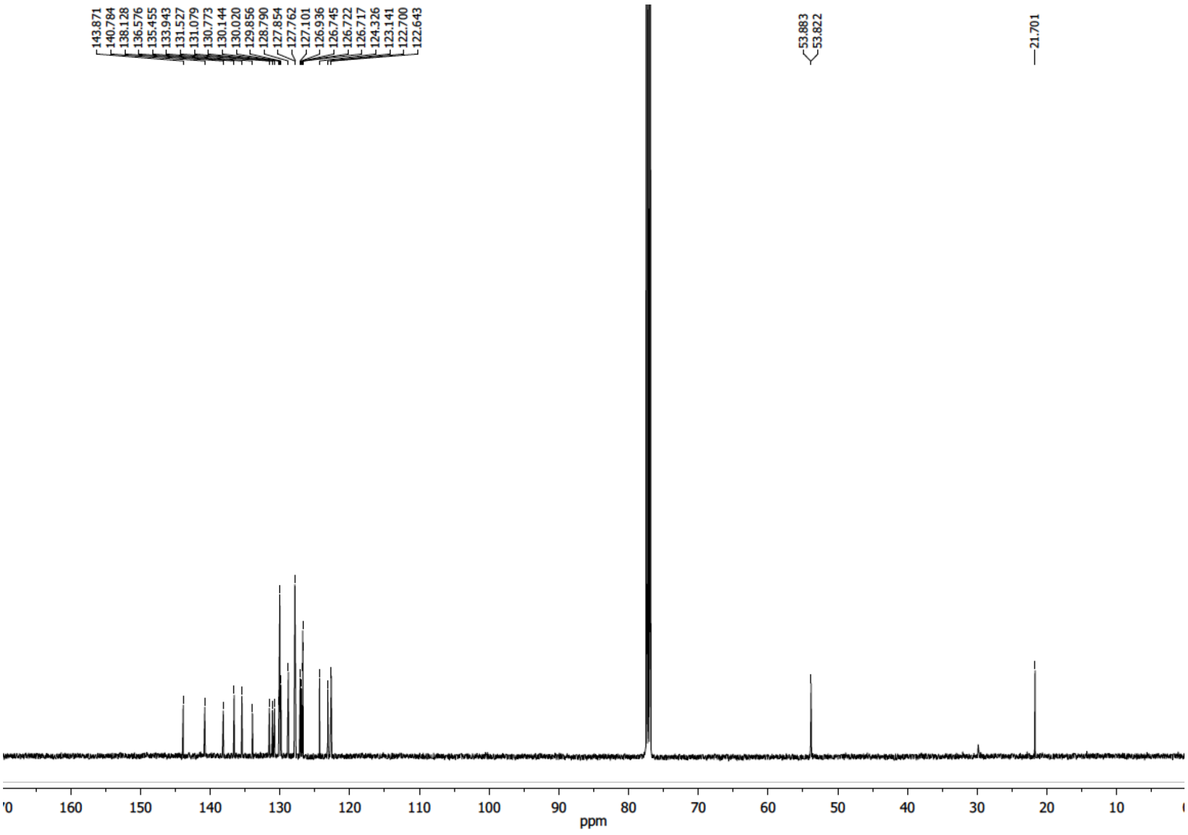

Figure S33. $^{13}C\{^{1}H\}$ NMR spectrum ($CDCl_3$)

2-Tosyl-5-(trimethylsilyl)isoindoline (6l)

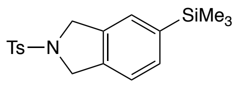

*R*f: 0.84 petroleum ether/AcOEt: 7/3 v/v; isolated yield 35 % (12 mg). ^{1}H NMR (500 MHz, $CDCl_3$): δ = 7.77 (d, 2H, arom CH, $^{3}J_{HH}$ = 8.0 Hz), 7.39 (d, 1H, arom CH, $^{3}J_{HH}$ = 7.5 Hz), 7.32-7.29 (m, 3H, arom CH), 7.16 (d, 1H, arom CH, $^{3}J_{HH}$ = 7.5 Hz), 4.62 (d, 2H, NCH_2, $^{4}J_{HH}$ = 2.5 Hz), 4.61 (d, 2H, NCH_2, $^{4}J_{HH}$ = 2.5 Hz), 2.40 (s, 3H, CH_3 of tosyl), -0.23 (s, 9H, $Si(CH_3)_3$); $^{13}C\{^{1}H\}$ NMR (126 MHz, $CDCl_3$): δ = 143.79, 140.48, 136.94, 135.75, 132.81 (5s, arom Cquat), 133.80, 129.94, 127.77, 127.54, 122.19 (5s, arom CH), 53.92 (s, NCH_2), 53.79 (s, NCH_2), 21.64 (s, CH_3 of tosyl), -0.99 (s, $Si(CH_3)_3$) ppm. MS (ESI-MS): m/z = 346.1290 $[M + H]^+$ (calcd. for $C_{18}H_{24}NSiSO_2$: 346.1292).

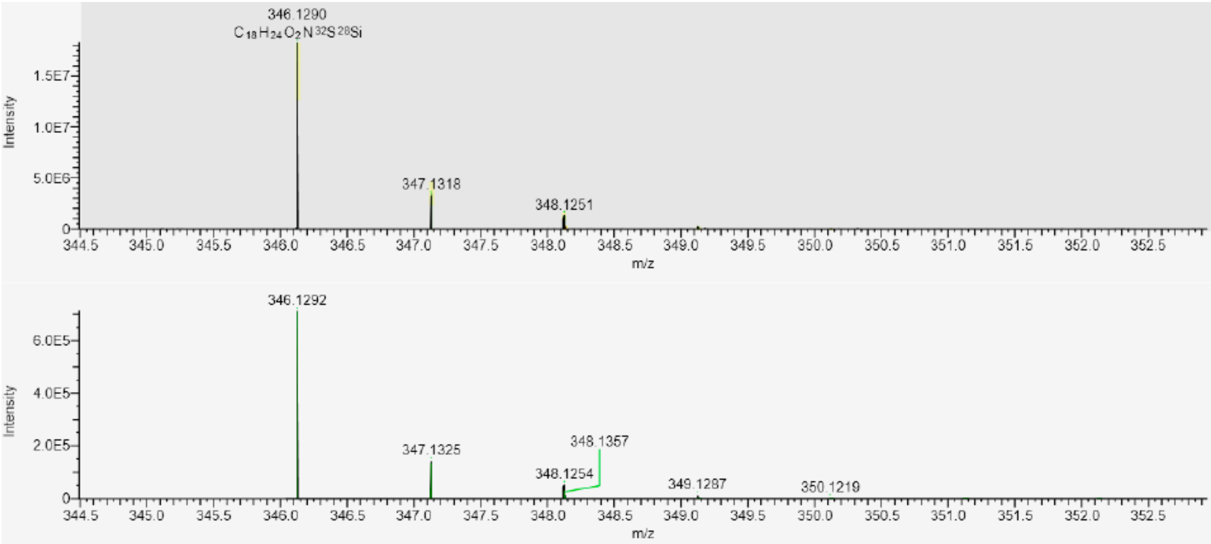

Figure S34. Mass spectrum (ESI-MS)
exp. spectrum (top); calc. spectrum (bottom) for $C_{18}H_{24}NSiSO_2$ $[M + H]^+$

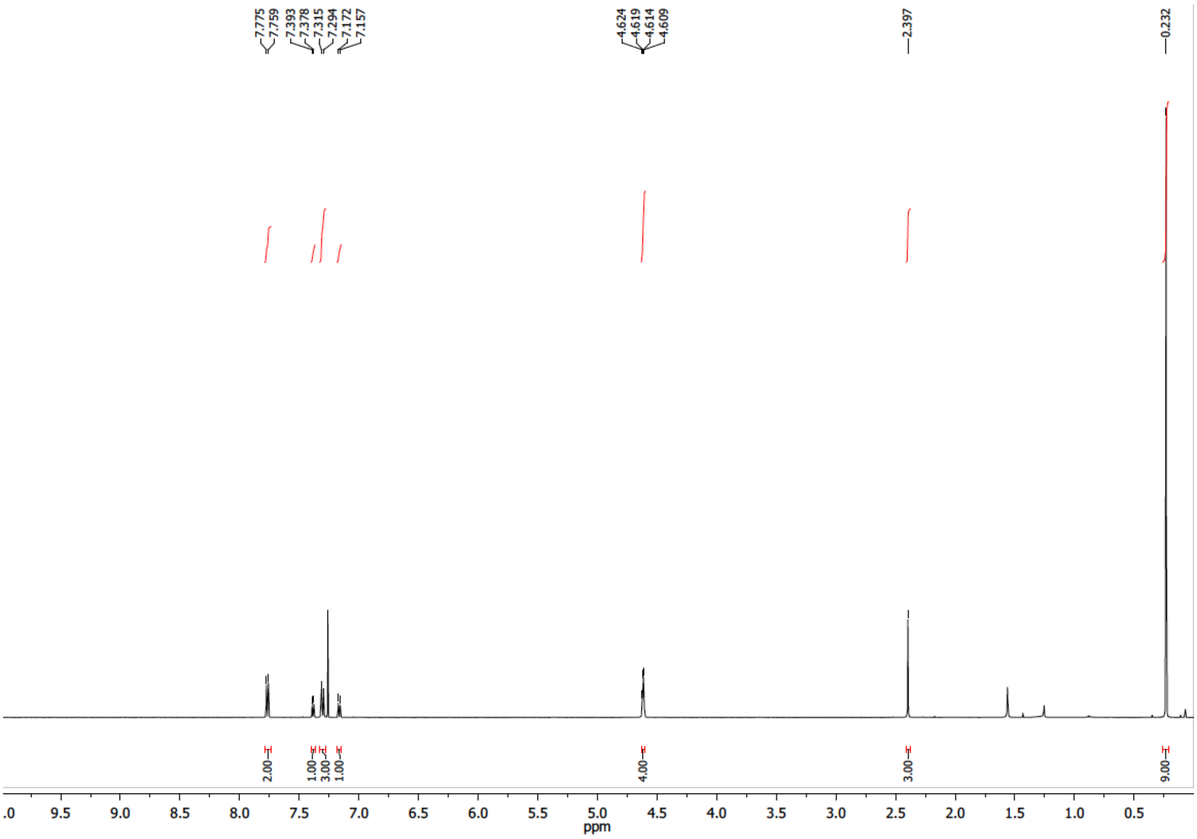

Figure S35. ^{1}H NMR spectrum ($CDCl_3$)

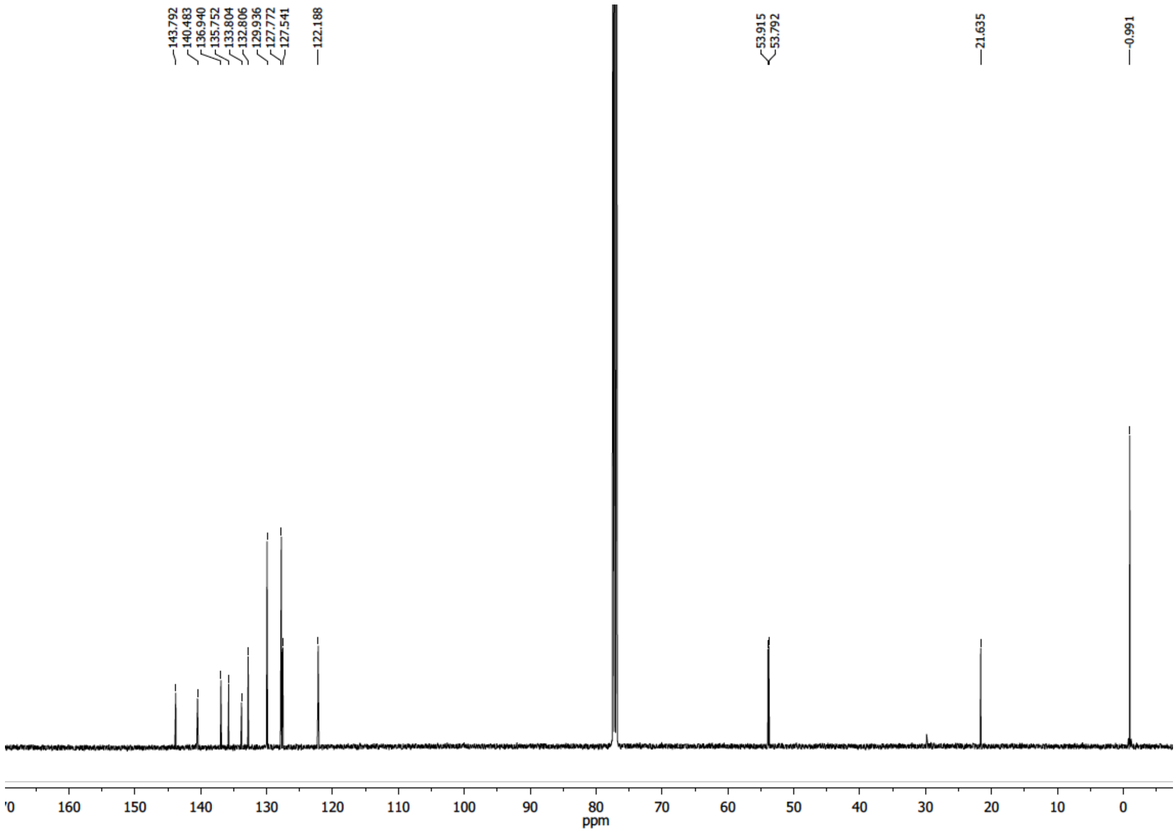

Figure S36. $^{13}C\{^{1}H\}$ NMR spectrum ($CDCl_3$)

(2-Tosylisoindolin-5-yl)methanol (6m)

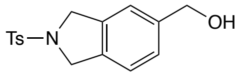

Rf: 0.12 petroleum ether/AcOEt: 7/3 v/v; isolated yield 79 % (31 mg). ^{1}H NMR (500 MHz, $CDCl_3$): δ = 7.76 (d, 2H, arom CH, $^{3}J_{HH}$ = 8.0 Hz), 7.31 (d, 2H, arom CH, $^{3}J_{HH}$ = 8.0 Hz), 7.23 (dd, 1H, arom CH, $^{3}J_{HH}$ = 7.5 Hz, $^{4}J_{HH}$ = 1.5 Hz), 7.19 (brs, 1H, arom CH), 7.14 (d, 1H, arom CH, $^{3}J_{HH}$ = 8.0 Hz), 4.65 (s, 2H, CH_2OH), 4.60 (brs, 2H, NCH_2), 4.60 (brs, 2H, NCH_2), 2.40 (s, 3H, CH_3 of tosyl); $^{13}C\{^{1}H\}$ NMR (126 MHz, $CDCl_3$): δ = 143.86, 140.95, 136.70, 135.63, 133.82 (5s, arom Cquat), 129.97, 127.73, 126.71, 122.84, 121.35 (5s, arom CH), 65.07 (s, CH_2OH), 53.77 (s, NCH_2), 53.68 (s, NCH_2), 21.65 (s, CH_3 of tosyl) ppm. Consistent with the literature data H. Kinoshita, H. Shinokubo, K. Oshima, *J. Am. Chem. Soc.* **2003**, *125*, 7784-7785.

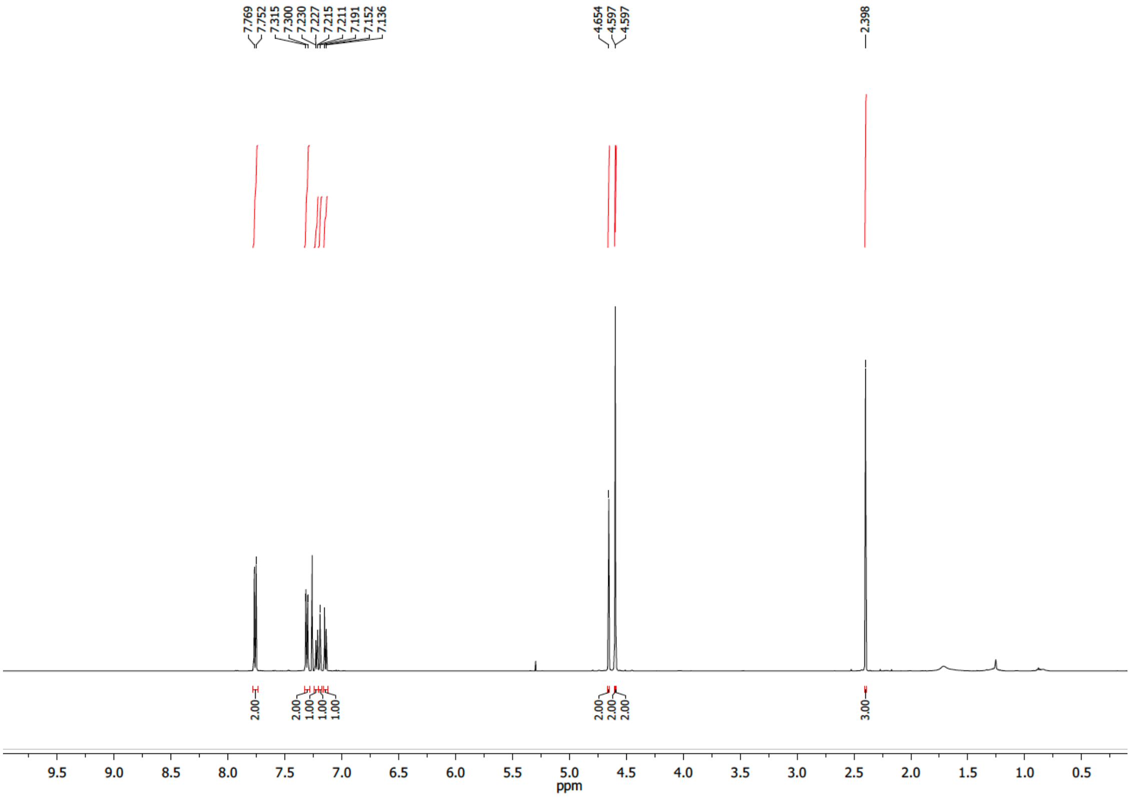

Figure S37. ^{1}H NMR spectrum ($CDCl_3$)

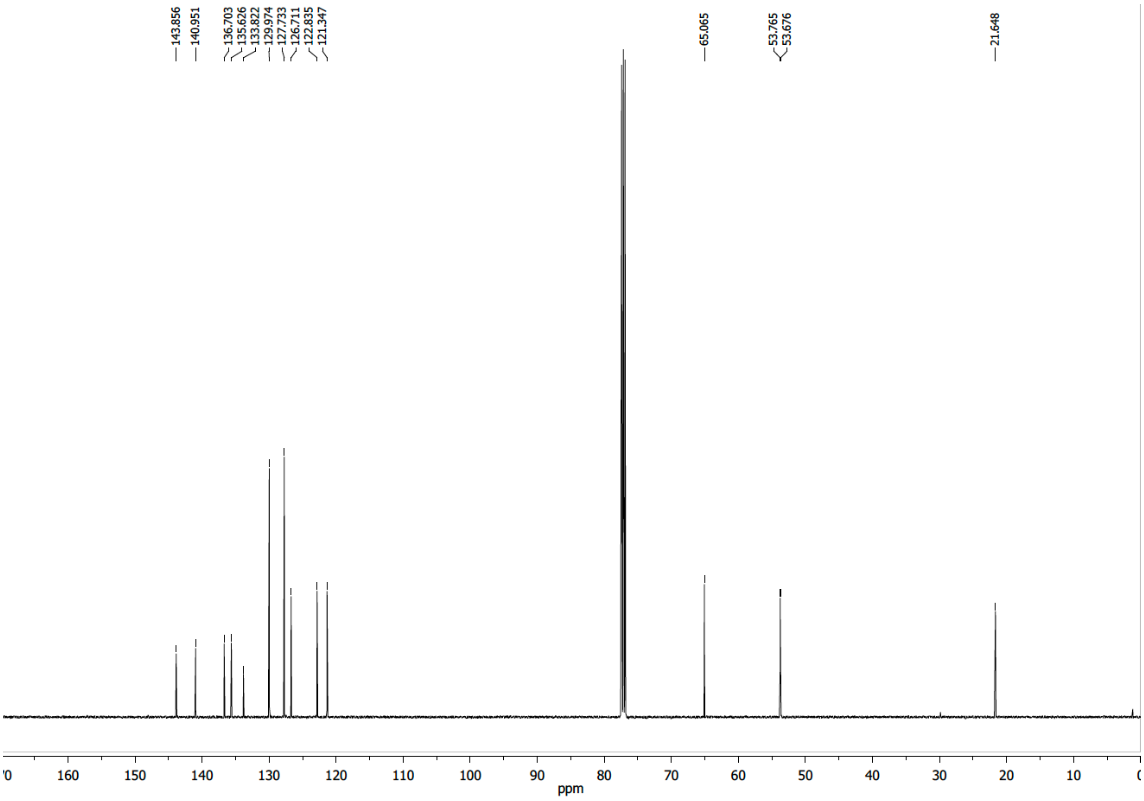

Figure S38. $^{13}C\{^{1}H\}$ NMR spectrum ($CDCl_3$)

4-Methyl-*N*-(prop-2-yn-1-yl)-*N*-((2-tosylisoindolin-5-yl)methyl)benzenesulfonamide (7)

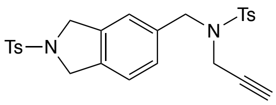

Rf: 0.30 petroleum ether/AcOEt: 7/3 v/v. ^{1}H NMR (500 MHz, $CDCl_3$): δ = 7.77 (d, 2H, arom CH, $^{3}J_{HH}$ = 8.0 Hz), 7.76 (d, 2H, arom CH, $^{3}J_{HH}$ = 8.5 Hz), 7.32 (d, 2H, arom CH, $^{3}J_{HH}$ = 7.5 Hz), 7.32 (d, 2H, arom CH, $^{3}J_{HH}$ = 7.5 Hz), 7.22 (dd, 1H, arom CH, $^{3}J_{HH}$ = 8.0 Hz, $^{4}J_{HH}$ = 2.0 Hz), 7.18 (brs, 1H, arom CH), 7.13 (d, 1H, arom. CH, $^{3}J_{HH}$ = 8.0 Hz), 4.60 (d, 2H, NCH_2 of NC_4H_4, $^{4}J_{HH}$ = 3.0 Hz), 4.59 (d, 2H, NCH_2 of NC_4H_4, $^{4}J_{HH}$ = 3.0 Hz), 4.30 (s, 2H, NCH_2 of $NCH_2C_6H_3$), 3.90 (d, 2H, NCH_2 of $CH_2C{\equiv}CH$, $^{4}J_{HH}$ = 2.5 Hz), 2.44 (s, CH_3 of tosyl), 2.40 (s, CH_3 of tosyl), 2.00 (t, 1H, $CH_2C{\equiv}C\mathit{H}$, $^{4}J_{HH}$ = 2.5 Hz); ^{13}C{^{1}H} NMR (126 MHz, $CDCl_3$): δ = 143.92, 143.87, 137.03, 136.35, 136.00, 135.11, 133.85 (7s, arom Cquat), 130.00, 129.71, 128.47, 127.94, 127.78, 123.11, 123.01 (7s, arom CH), 76.22 (s, Cquat of $\mathit{C}{\equiv}CH$), 74.38 (s, CH of $C{\equiv}\mathit{C}H$), 53.66 (s, CH_2 of NC_4H_4), 53.64 (s, CH_2 of NC_4H_4), 49.63 (s, CH_2 of $N\mathit{C}H_2C_6H_3$), 35.77 (s, CH_2 of $\mathit{C}H_2C{\equiv}CH$), 21.73 (s, CH_3 of tosyl), 21.67 (s, CH_3 of tosyl) ppm. Consistent with the literature data D. J. Nasrallah, M. P. Croatt, *Eur. J. Org. Chem.* **2014**, *2014*, 3767-3772.

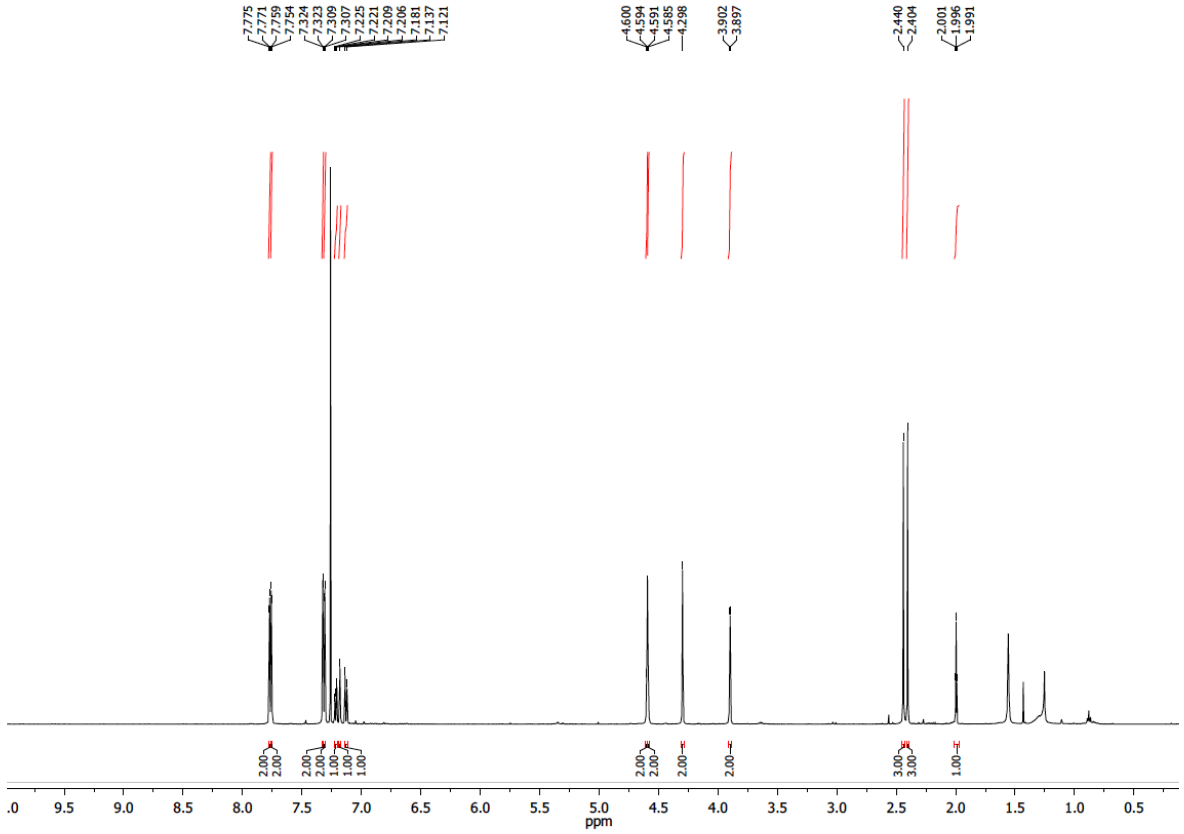

Figure S39. ^{1}H NMR spectrum ($CDCl_3$)

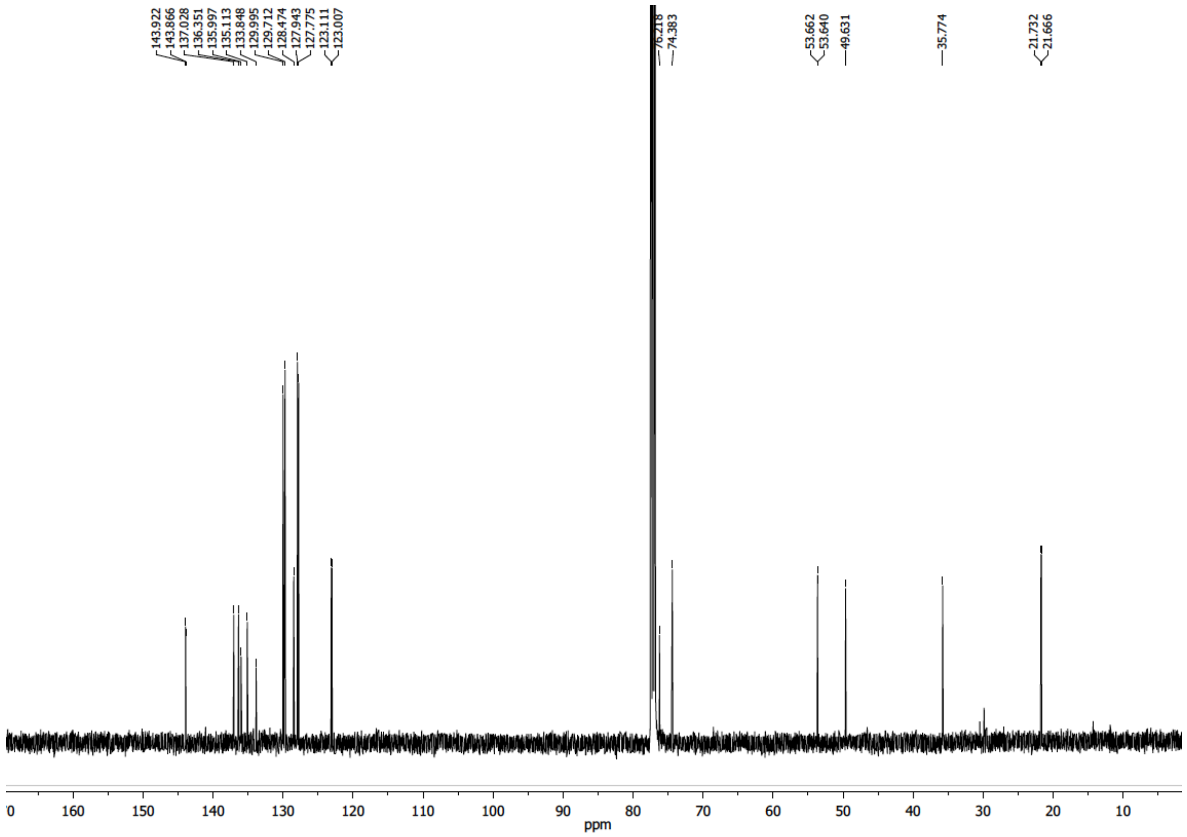

Figure S40. $^{13}C\{^{1}H\}$ NMR spectrum ($CDCl_3$)

4-Methyl-*N*,*N*-bis((2-tosylisoindolin-5-yl)methyl)benzenesulfonamide (8)

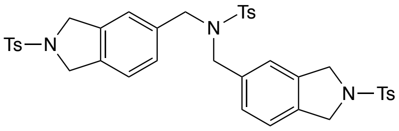

Rf: 0.11 petroleum ether/AcOEt: 7/3 v/v. ^{1}H NMR (300 MHz, $CDCl_3$): δ = 7.76 (d, 4H, arom CH, $^3J_{HH}$ = 8.4 Hz), 7.67 (d, 2H, arom CH, $^3J_{HH}$ = 8.4 Hz), 7.33 (d, 4H, arom CH, $^3J_{HH}$ = 8.4 Hz), 7.28 (d, 2H, arom CH, $^3J_{HH}$ = 8.4 Hz), 6.97 (d, 2H, arom CH, $^3J_{HH}$ = 8.1 Hz), 6.85 (d, 2H, arom CH, $^3J_{HH}$ = 8.1 Hz), 6.81 (s, 2H, arom CH), 4.52 (s, 4H, NCH_2), 4.46 (s, 4H, NCH_2), 4.21 (s, 4H, NCH_2), 2.44 (s, 3H, CH_3 of tosyl), 2.41 (s, 6H, CH_3 of tosyl); $^{13}C\{^1H\}$ NMR (126 MHz, $CDCl_3$): δ = 143.92, 143.77, 137.53, 136.74, 135.88, 135.68, 133.83 (7s, arom Cquat), 130.00, 129.89, 128.13, 127.75, 127.29, 122.75, 122.65 (7s, arom CH), 53.52 (s, NCH_2), 53.49 (s, NCH_2), 50.53 (s, NCH_2), 21.68 (s, CH_3 of tosyl), 21.66 (s, CH_3 of tosyl) ppm. Consistent with the literature data D. J. Nasrallah, M. P. Croatt, *Eur. J. Org. Chem.* **2014**, *2014*, 3767-3772.

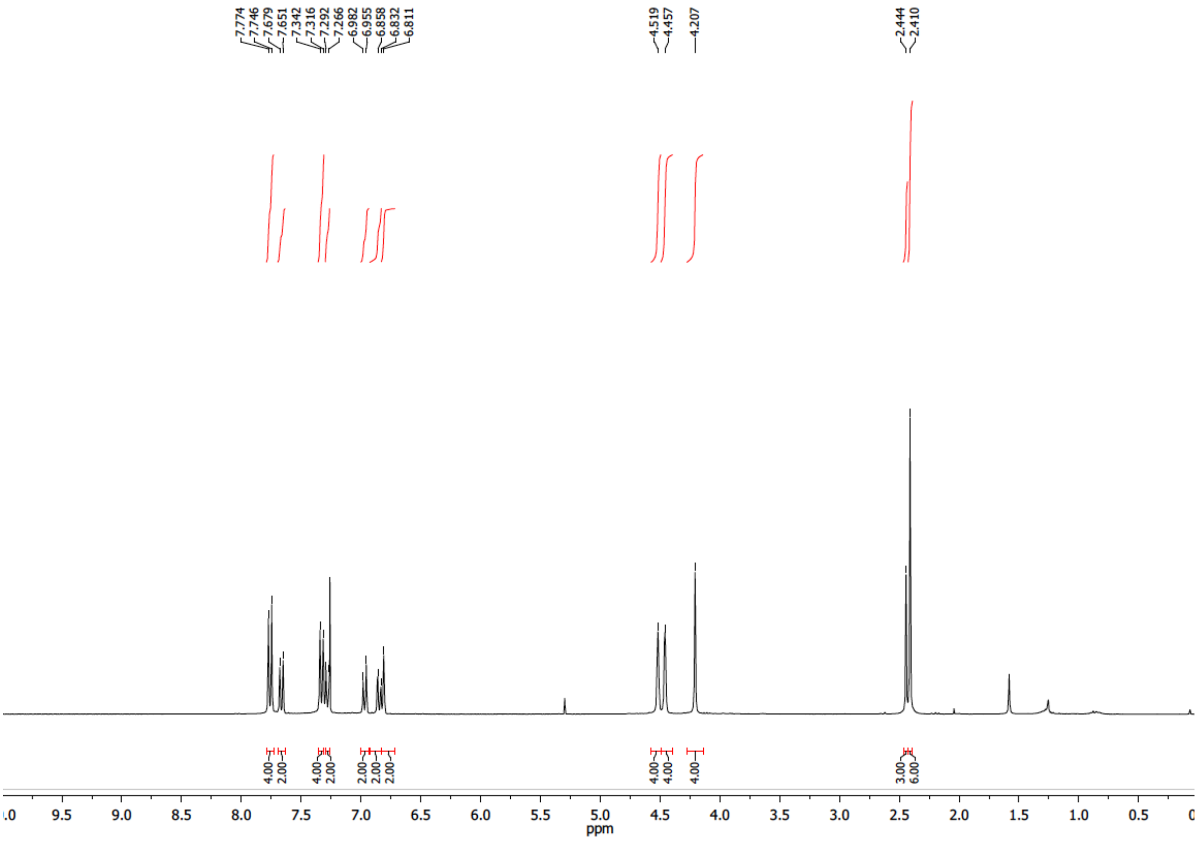

Figure S41. ^{1}H NMR spectrum ($CDCl_3$)

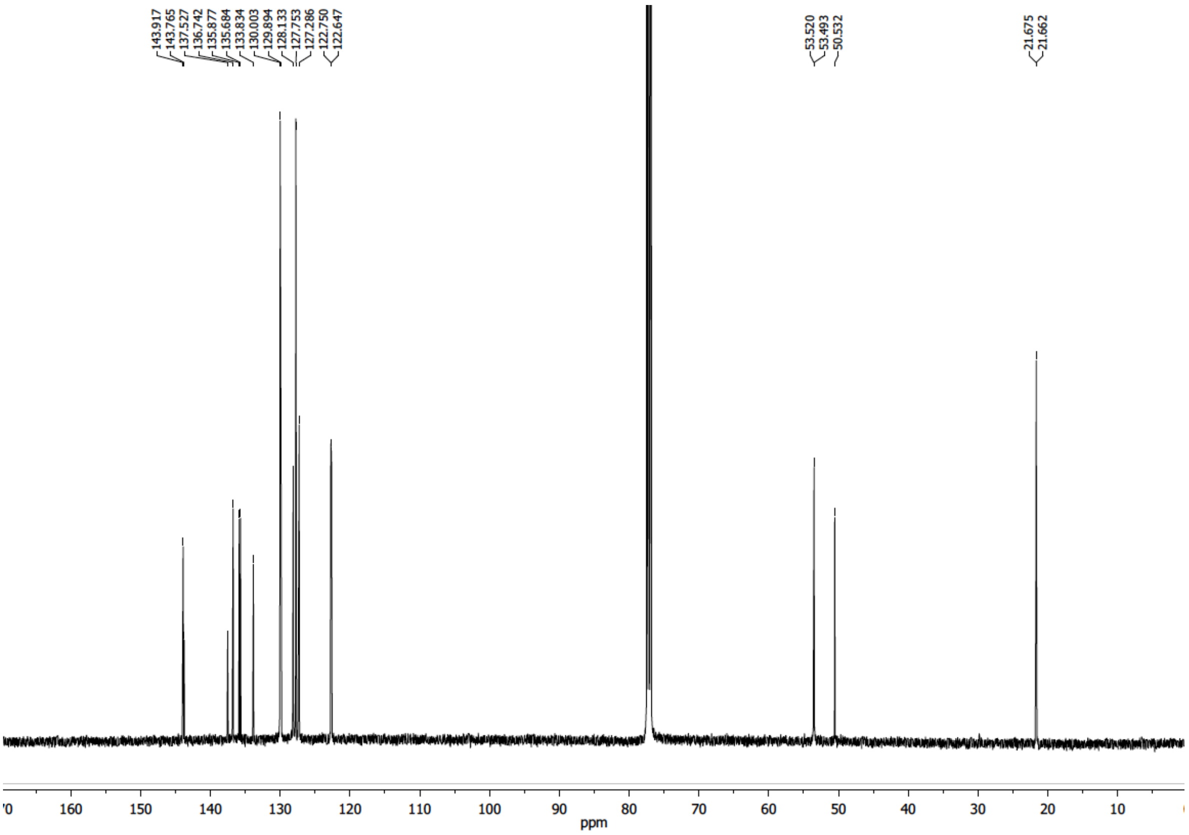

Figure S42. $^{13}C\{^{1}H\}$ NMR spectrum ($CDCl_3$)
